# Data-driven discovery of Green’s functions with human-understandable deep learning

**DOI:** 10.1038/s41598-022-08745-5

**Published:** 2022-03-22

**Authors:** Nicolas Boullé, Christopher J. Earls, Alex Townsend

**Affiliations:** 1 Mathematical Institute, University of Oxford, Oxford, OX2 6GG UK; 2 Center for Applied Mathematics, Cornell University, Ithaca, NY 14853 USA; 3 School of Civil and Environmental Engineering, Cornell University, Ithaca, NY 14853 USA; 4 Department of Mathematics, Cornell University, Ithaca, NY 14853 USA

**Keywords:** Mathematics and computing, Applied mathematics, Computational science, Software, Engineering

## Abstract

There is an opportunity for deep learning to revolutionize science and technology by revealing its findings in a human interpretable manner. To do this, we develop a novel data-driven approach for creating a human–machine partnership to accelerate scientific discovery. By collecting physical system responses under excitations drawn from a Gaussian process, we train rational neural networks to learn Green’s functions of hidden linear partial differential equations. These functions reveal human-understandable properties and features, such as linear conservation laws and symmetries, along with shock and singularity locations, boundary effects, and dominant modes. We illustrate the technique on several examples and capture a range of physics, including advection–diffusion, viscous shocks, and Stokes flow in a lid-driven cavity.

## Introduction

Deep learning (DL) holds promise as a scientific tool for discovering elusive patterns within the natural and technological world^1,2^. These patterns hint at undiscovered partial differential equations (PDEs) that describe governing phenomena within biology, fluid dynamics, and physics. From sparse and noisy laboratory observations, we aim to learn mechanistic laws of nature^3,4^. Recently, scientific computing and machine learning have successfully converged on PDE discovery^5–8^, PDE learning^9–14^, and symbolic regression^15,16^ as promising means for applying machine learning to scientific investigations. These methods attempt to discover the coefficients of a PDE model or learn the operator that maps excitations to system responses. The recent DL techniques addressing the latter problem are based on approximating the solution operator associated with a PDE by a neural network^9–13^. While excellent for solving PDEs, we consider them as “black box” and focus here on a data-driven strategy that improves human understanding of the governing PDE model.

In contrast, we offer a radically different, alternative approach that is backed by theory^17^ and infuse an interpretation in the model by learning well-understood mathematical objects that imply underlying physical laws. We devise a DL method, employed for learning the Green’s functions^18^ associated with unknown governing linear PDEs, and train the neural networks (NNs) by collecting physical system responses from random excitation functions drawn from a Gaussian process. The empirically derived Green’s functions relate the system’s response (or PDE solution) to a forcing term, and can then be used as a fast reduced-order PDE solver. The existing Graph Kernel Network^12^ and DeepGreen^11^ techniques also aim to learn solution operators of PDEs based on Green’s functions. While they show competitive performance in predicting the solution of the PDE for new forcing functions, they fail to capture Green’s functions accurately, which makes the extraction of qualitative and quantitative features of the physical system challenging.

Our primary objective is to study the discovered Green’s functions for clues regarding the physical properties of the observed systems. Our approach relies on a novel and adaptive neural network architecture called a rational neural network^19^, which has higher approximation power than standard networks and carries human-understandable features of the PDE, such as shock and singularity locations.

In this paper, we use techniques from deep learning to discover the Green’s function of linear differential equations $\mathcal {L}u = f$ from input–output pairs (*f*, *u*), as opposed to directly learning $\mathcal {L}$, or model parameters. In this sense, our approach is agnostic to the forward PDE model, but nonetheless offers insights into its physical properties. There are several advantages to learning the Green’s function. First, once the Green’s function is learned by a neural network (NN), it is possible to compute the solution, *u*, for a new forcing term, *f*, by evaluating an integral (see Eq. (1)); which is more efficient than training a new NN. Second, the Green’s function associated with $\mathcal {L}$ contains information about the operator, $\mathcal {L}$, and the type of boundary constraints that are imposed; which helps uncover mechanistic understanding from experimental data. Finally, it is easier to train NNs to approximate Green’s functions, which are square-integrable functions under sufficient regularity conditions^18,20,21^, than trying to approximate the action of the linear differential operator, $\mathcal {L}$, which is not bounded^22^. Also, any prior mathematical and physical knowledge of the operator, $\mathcal {L}$, can be exploited in the design of the NN architecture, which could enforce a particular structure such as symmetry of the Green’s function.

## Results

### Deep learning Green’s functions

**Figure 1:**
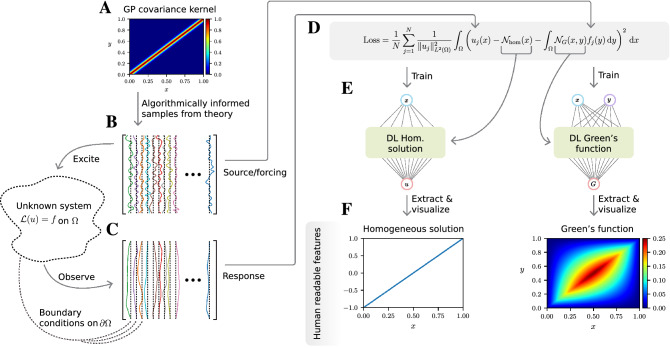
Schematic of our DL method for learning Green’s functions from input-output pairs. (**A**) The covariance kernel of the Gaussian process (GP), which is used to generate excitations. (**B**) The random excitations and the system’s response are recorded (**C**). (**D**) A loss function is minimized to train rational NNs (**E**). (**F**) The learned Green’s function and homogeneous solution are visualized by sampling the NNs.

Our DL approach (see Fig. 1) begins with excitations (or forcing terms), $\{f_j\}_{j=1}^N$, sampled from a Gaussian process (GP) having a carefully designed covariance kernel^17^, and corresponding system responses, $\{u_j\}_{j=1}^N$. It is postulated that there is an unknown linearized governing PDE so that $\mathcal {L}u_j = f_j$. The selection of random forcing terms is theoretically justified^17^ and enables us to learn the dominant eigenmodes of the solution operator, using only a small number, *N*, of training pairs. The Green’s function, *G*, and homogeneous solution, $u_{\text {hom}}$, which encodes the boundary conditions associated with the PDE, satisfy

1 $$\begin{aligned} u_j(x) = \int _{\Omega }G(x,y)f_j(y)\,d y + u_{\text {hom}}(x),\qquad x\in \Omega , \end{aligned}$$

and are approximated by two rational neural networks: $\mathcal {N}_G$ and $\mathcal {N}_{\text {hom}}$.

A rational NN consists of a NN with trainable rational activation functions whose coefficients are learned simultaneously with the weights and biases of the network. Rational NNs have better approximation properties than standard NNs^19^, both in theory and in practice, which makes them ideal for the present application. The parameters of the NNs representing the Green’s function and homogeneous solution are simultaneously learned through minimization of the loss function displayed in Fig. 1D (Supplementary Material, Sect. 2). We discretize the integrals in the loss function at the specified measurement locations $\{x_i\}_{i=1}^{N_u}$, within the domain, $\Omega$, and forcing term sample points, $\{y_i\}_{i=1}^{N_f}$, respectively, using a quadrature rule.

In the Supplementary Material, Fig. S4, we also present numerical results obtained from sparse training data, or noisy spatial measurements, which demonstrate the robustness of our method (Supplementary Material, Sect. 4). Additionally, our DL technique is data-driven and requires minimal by-hand parameter tuning. In fact, all the numerical examples described here and in the Supplementary Material are performed using a unique rational NN architecture, initialization procedure, and optimization algorithm.

### Human-understandable features

**Figure 2:**
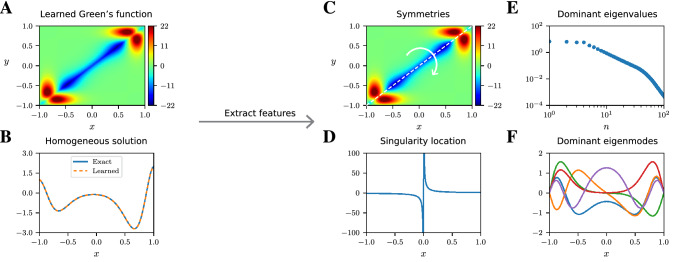
Feature extraction from learned Green’s functions. The NNs for the learned Green’s function (**A**) and homogeneous solution (**B**) enable the extraction of qualitative and quantitative features associated with the differential operator. For example, the symmetries in the Green’s function reveal PDE invariances (**C**), poles of rational NNs identify singularity type and location (**D**), the dominant eigenvalues (**E**) and eigenmodes (**F**) of the learned Green’s function are related to the eigenvalues and eigenmodes of the differential operator.

The trained NNs contain both the desired Green’s function and homogeneous solution, which we evaluate and visualize to glean novel insights concerning the underlying, governing PDE (Fig. 2). In this way, we achieve one part of our human interpretation goal: finding a link between the properties of the Green’s function and that of the underlying differential operator and solution constraints.

As an example, if the Green’s function is symmetric, i.e., $G(x,y)=G(y,x)$ for all $x,y\in \Omega$, then the operator $\mathcal {L}$ is self-adjoint. Another aspect of human interpretability is that the poles of the trained rational NN tend to cluster in a way that reveal the location and type of singularities in the homogeneous solution. Finally, there is a direct correspondence between the dominant eigenmodes and eigenvalues (as well as the singular vectors and singular values) of the learned Green’s function and those of the differential operator. The correspondence gives insight into the important eigenmodes that govern the system’s behavior (Supplementary Material, Sect. 5).

**Figure 3:**
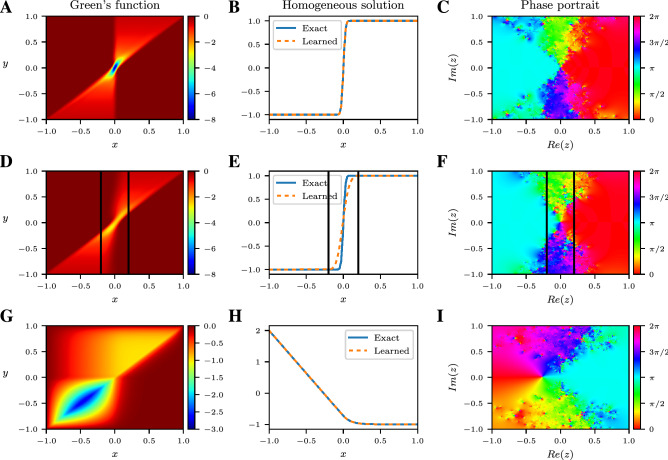
Green’s functions learned by rational neural networks. (**A**) Green’s function of a differential operator with a viscous shock at $x=0$, learned by a rational NN. (**B**) Learned and exact (computed by a classical spectral method) homogeneous solution to the differential equation with zero forcing term. (**C**) Phase portrait of the homogeneous rational NN evaluated on the complex plane. (**D–F**) Similar to (**A–C**), but without any system’s response measurements in $x\in [-\, 0.2,0.2]$ (see vertical black lines) near the shock. (**G**) Learned Green’s function and homogeneous solution (**H**) of an advection–diffusion operator with advection occurring for $x\ge 0$. (**I**) Phase portrait of the homogeneous NN on the complex plane.

### Numerical examples

As a first example, we consider a second-order differential operator having suitable variable coefficients to model a viscous shock at $x=0$^23^. The system’s responses are obtained by solving the PDE, with Dirichlet boundary conditions, using a spectral numerical solver for each of the $N = 100$ random forcing terms, sampled from a GP having a squared-exponential covariance kernel^17^. The learned Green’s function is displayed in Fig. 3A and satisfies the following symmetry relation: $G(x,y) = G(- x, - y)$, indicating the presence of a reflective symmetry group within the underlying PDE. Indeed, if *u* is a solution to $\mathcal {L}u=f$ with homogeneous boundary conditions, then $u(- x)$ is a solution to $\mathcal {L}v=f(- x)$. We also observe in Fig. 3B,C that the homogeneous solution is accurately captured and that the poles of the homogeneous rational NN cluster near the real axis around $x=0$: the location of the singularity induced by the shock (Supplementary Material, Sect. 5E).

Next, we reproduce the same viscous shock numerical experiment, except that this time we remove measurements of the system’s response from the training dataset in the interval $[-\,0.2, 0.2]$: adjacent to the shock front. By comparing Fig. 3A–F, we find that the Green’s function and homogeneous solution, learned by the rational NNs, may not be affected in the region outside of the interval with missing data. In some cases, the NNs can still accurately capture the main features of the Green’s function and homogeneous solution in the region lacking measurements. The robustness of our method to noise perturbation and corrupted or missing data is of significant interest and promising for real applications with experimental data.

We next apply our DL method to discover the Green’s function and homogeneous solution of an advection–diffusion operator, where the advection is dominant only within the right half of the domain. The output of the Green’s function NN is plotted in Fig. 3G, where we observe the disparate spatial behaviors of the dominant physical mechanisms. This can be recognized when observing the restriction of the Green’s function to the subdomain $[- 1, 0]\times [- 1, 0]$, where the observed solution is reminiscent of the Green’s function for the Laplacian; thus indicating that the PDE is diffusive on the left half of the domain. Similarly, the restriction of the learned Green’s function to $[0, 1]\times [0, 1]$ is characteristic of advection.

In Fig. 3H,I, we display the homogeneous solution NN, along with the phase of the rational NN, evaluated on the complex plane. The agreement between the exact and learned homogeneous solution illustrates the ability of the DL method to accurately capture the behavior of a system within “multiphysics” contexts. The choice of rational NNs is crucial here: to deepen our understanding of the system, as the poles of the homogeneous rational NN characterize the location and type of singularities in the homogeneous solution. Here the change in behavior of the differential operator from diffusion to advection is delineated by the location of the poles of the rational NN.

### Nonlinear and vector-valued equations

**Figure 4:**
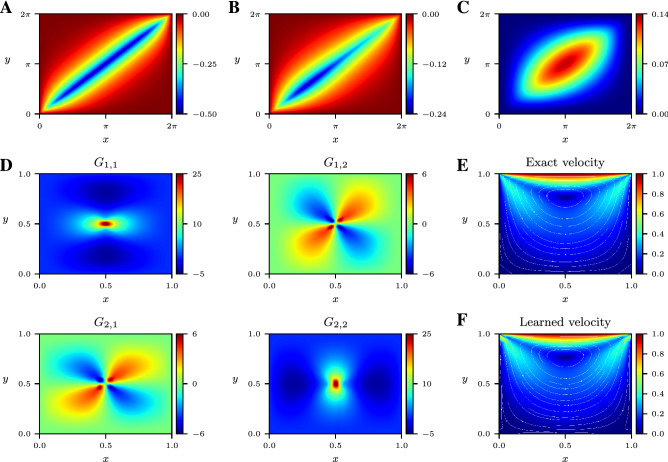
Linearized models and Stokes flow. (**A–C**) Green’s functions of three differential operators: Helmholtz, Sturm–Liouville, and biharmonic, with cubic nonlinearity. (**D**) Matrix of Green’s functions of a two-dimensional Stokes flow in a lid-driven cavity, evaluated at a two-dimensional slice. Velocity magnitude and streamlines of the exact (**E**) and learned (**F**) homogeneous solution to the Stokes equations with zero applied body force.

We can also discover Green’s functions from forcing terms and concomitant solutions to nonlinear differential equations possessing semi-dominant linearity. In Fig. 4A–C, we visualize the Green’s function NNs of three operators with cubic nonlinearity considered in Ref.^11^. The nonlinearity does not prevent our method from discovering a Green’s function of an approximate linear model, from which one can understand features such as symmetry and boundary conditions. This property is crucial for tackling time-dependent problems, where the present technique may be extended and applied to uncover linear propagators.

Finally, we consider a Stokes flow in a two-dimensional lid-driven cavity to emphasize the ability of our method to handle systems of differential equations in two dimensions. In this context, the relation between the system’s responses and the forcing terms can be expressed using a Green’s matrix, which consists of a two-by-two matrix of Green’s functions and whose components reveal features of the underlying system such as symmetry and coupling (Fig. 4D, Supplementary Material, Sects. 7, 8D). Figure 4E,F illustrate that the homogeneous solution to the Stokes equation is accurately captured by the homogeneous rational NN, despite the corner singularities and coarse measurement grid (Supplementary Material, Sect. 8D).

## Discussion

Contrary to existing works in the literature^10–12,24^, our primary aim is to uncover mechanistic understanding from input–output data using a human-understandable representation of the underlying, but hidden, differential operator. This representation takes the form of a rational NN^19^ for the Green’s function. We extensively described all the physical features of the operator that can be extracted and discovered from the learned Green’s function and homogeneous solutions, such as linear conservation laws, symmetries, shock front and singularity locations, boundary conditions, and dominant modes.

The DL method for learning Green’s functions of linear differential operators naturally extends to the case of three spatial dimensions but these systems are more challenging due to the GPU memory demands required to represent the six-dimensional inputs used to train the NN representing the Green’s function. However, alternative optimization algorithms than the one used in this paper and described in “Methods” section, such as mini-batch optimization^25,26^, may be employed to alleviate the computational expense of the training procedure.

While our method is demonstrated on linear differential operators, it can be extended to nonlinear, time-dependent problems that can be linearized using an implicit-explicit time-stepping scheme^27,28^ or an iterative method^29^. This process allows us to learn the Green’s functions of linear time propagators and understand physical behavior in time-dependent problems from input-output data such as the time-dependent Schrödinger equation (Supplementary Material, Sect. 9). The numerical experiments conducted in Fig. 4A–C highlight that our approach can discover Green’s functions of linearizations of nonlinear differential operators.

Our deep learning method for learning Green’s functions and extracting human-understandable properties of partial differential equations benefits from the adaptivity of rational neural networks and its support for qualitative feature detection and interpretation. We successfully tested our approach with noisy and sparse measurements as training data (Supplementary Material, Sect. 4). The design of our applied network architectures, and covariance kernel used to generate the system forcing is guided by rigorous theoretical statements^17,19^ that offer performance guarantees. This shows that our proposed deep learning method may be used to discover new mechanistic understanding with machine learning.

## Methods

### Green’s functions

We consider linear differential operators, $\mathcal {L}$, defined on a bounded domain $\Omega \subset \mathbb {R}^d$, where $d\in \{1,2,3\}$ denotes the spatial dimension. The aim of our method is to discover properties of the operator, $\mathcal {L}$, using *N* input-output pairs $\{(f_j,u_j)\}_{j=1}^N$, consisting of forcing functions, $f_j:\Omega \rightarrow \mathbb {R}$, and system responses, $u_j:\Omega \rightarrow \mathbb {R}$, which are solutions to the following equation:

2 $$\begin{aligned} \mathcal {L}u_j = f_j, \qquad \mathcal {D}(u_j,\Omega ) = g, \end{aligned}$$

where $\mathcal {D}$ is a linear operator acting on the solutions, *u*, and the domain, $\Omega$; with *g* being the constraint. We assume that the forcing terms have sufficient regularity, and that the operator, $\mathcal {D}$, is a constraint so that Eq. (2) has a unique solution^18^. An example of constraint is the imposition of homogeneous Dirichlet boundary conditions on the solutions: $\mathcal {D}(u_j,\Omega ) := u_{j}|_{\partial \Omega }=0$. Note that boundary conditions, integral conditions, jump conditions, or non-standard constraints, are all possible (Supplementary Material, Sect. 5A).

A Green’s function^18,30–32^ of the operator, $\mathcal {L}$, is defined as the solution to the following equation:

$$\begin{aligned} \mathcal {L}G(x,y) = \delta (y-x),\qquad x,y\in \Omega , \end{aligned}$$

where $\mathcal {L}$ is acting on the function $x\mapsto G(x,y)$ for fixed $y\in \Omega$, and $\delta (\cdot )$ denotes the Dirac delta function. The Green’s function is well-defined and unique under mild conditions on $\mathcal {L}$, and suitable solution constraints imposed via an operator, $\mathcal {D}$ (see Eq. (2))^18^. Moreover, if (*f*, *u*) is an input-output pair, satisfying Eq. (2) with $g=0$, then

$$\begin{aligned} u(x) = \int _{\Omega }G(x,y)f(y)\,d y, \qquad x\in \Omega . \end{aligned}$$

Therefore, the Green’s function associated with $\mathcal {L}$ can be thought of as the right inverse of $\mathcal {L}$.

Let $u_{\text {hom}}$ be the homogeneous solution to (2), so that

$$\begin{aligned} \mathcal {L}u_{\text {hom}} = 0, \qquad \mathcal {D}(u_{\text {hom}},\Omega ) = g. \end{aligned}$$

Using superposition, we can construct solutions, $u_j$, to Eq. (2) as $u_j = {\tilde{u}}_j+u_{\text {hom}}$, where ${\tilde{u}}_j$ satisfies

$$\begin{aligned} \mathcal {L}{\tilde{u}}_j = f_j, \qquad \mathcal {D}({\tilde{u}}_j,\Omega ) = 0. \end{aligned}$$

Then, the relation between the system’s response, $u_j$, and the forcing term, $f_j$, can be expressed via the Green’s function as

$$\begin{aligned} u_j(x) = \int _{\Omega }G(x,y)f_j(y)\,d y + u_{\text {hom}}(x),\qquad x\in \Omega . \end{aligned}$$

Therefore, we train two NNs: $\mathcal {N}_G:\Omega \times \Omega \rightarrow \mathbb {R}\cup \{\pm \infty \}$ and $\mathcal {N}_{\text {hom}}:\Omega \rightarrow \mathbb {R}$, to learn the Green’s function, and also the homogeneous solution associated with $\mathcal {L}$ and the constraint operator $\mathcal {D}$. Note that this procedure allows us to discover boundary conditions, or constraints, directly from the input–output data without imposing it in the loss function (which often results in training instabilities^33^).

### Rational neural networks

Rational NNs^19^ consist of NNs with adaptive rational activation functions $x\mapsto \sigma (x) = p(x)/q(x)$, where *p* and *q* are two polynomials, whose coefficients are trained at the same time as the other parameters of the networks, such as the weights and biases. These coefficients are shared between all the neurons in a given layer but generally differ between the network’s layers. This type of network was proven to have better approximation power than standard Rectified Linear Unit (ReLU) networks^34,35^, which means that they can approximate smooth functions more accurately with fewer layers and network parameters^19^. It is also observed in practice that rational NNs require fewer optimization steps and therefore can be more efficient to train than other activation functions^19^.

The NNs, $\mathcal {N}_G$ and $\mathcal {N}_{\text {hom}}$, which approximate the Green’s function and homogeneous solution associated with Eq. (2), respectively, are chosen to be rational NNs^19^ with 4 hidden layers and 50 neurons in each layer. We choose the polynomials, *p* and *q*, within the activation functions to be of degree 3 and 2, respectively, and initialize the coefficients of all the rational activation functions so that they are the best (3, 2) rational approximant to a ReLU (see the Supplementary Material of Ref.^19^ for details). The motivation is that the flexibility of the rational functions brings extra benefit in the training and accuracy over the ReLU activation function. We highlight that the increase in the number of trainable parameters, due to the adaptive rational activation functions, is only linear with respect to the number of layers and negligible compared to the total number of parameters in the network as:

$$\begin{aligned} \text {Number of rational coefficients} = 7 \times \text {number of hidden layers} = 28. \end{aligned}$$

The weight matrices of the NNs are initialized using Glorot normal initializer^36^, while the biases are initialized to zero.

Another advantage of rational NNs is the potential presence of poles, i.e., zeros of the polynomial *q*. While the initialization of the activation functions avoids training issues due to potential spurious poles, the poles can be exploited to learn physical features of the differential operator (Supplementary Material, Sect. 5E). Therefore, the architecture of the NNs also supports the aim of a human-understandable approach for learning PDEs. In higher dimensions, such as $d = 2$ or $d =3$, the Green’s function is not necessarily bounded along the diagonal, i.e., $\{(x,x),\, x\in \Omega \}$; thus making the poles of the rational NNs crucial.

Finally, we emphasize that the enhanced approximation properties of rational NNs^19^ make them ideal for learning Green’s functions and, more generally, approximating functions within regression problems. These networks may also be of benefit to other approaches for solving and learning PDEs with DL techniques, such as PINNs^37^, DeepGreen^11^, DeepONet^10^, Neural operator^12^, and Fourier neural operator^24^.

### Data generation

We create a training dataset, consisting of input-output functions, $\{(f_j\,u_j)\}$ for $1\le j \le N$, in three steps: (1) Generating the forcing terms by sampling random functions from a Gaussian process (GP), (2) Solving Eq. (2) for the generated forcing terms, and (3) Sampling the forcing terms, $f_j$, at the points $\{y_1,\ldots ,y_{N_f}\}\subset \Omega$ and the system’s responses, $u_j$, at $\{x_1,\ldots ,x_{N_u}\}\subset \Omega$. Here, $N_f$ and $N_u$ are the forcing and solution discretization sizes, respectively. We recommend that all the forcing terms are sampled on the same grid and similarly for the system’s responses. This minimizes the number of evaluations of $\mathcal {N}_G$ during the training phase and reduces the computational and memory costs of training.

The spatial locations of points $\{y_i\}$ and the forcing discretization size, $N_f$, are chosen arbitrarily to train the NNs as the forcing terms are assumed to be known over $\Omega$. In practice, the number, $N_u$, and location of the measurement points, $\{x_i\}$, are imposed by the nature of the experiment, or simulation, performed to measure the system’s response. When $\Omega$ is an interval, we always select $N_f=200$, $N_u=100$, and equally-spaced sampled points for the forcing and response functions. Further details on the training data generation are available in the Supplementary Material, Sect. 1. We then analyze the robustness of our method for learning Green’s functions with respect to the number and location of the measurement points in the Supplementary Material, Sect. 4.

### Neural network training

The NNs are implemented with single-precision floating-point format within the TensorFlow DL library^38^, and are trained (the numerical experiments are performed on a desktop computer with a Intel$^\text{\textregistered }$ Xeon$^\text{\textregistered }$ CPU E5-2667 v2 @ 3.30 GHz and a NVIDIA$^\text{\textregistered }$ Tesla$^\text{\textregistered }$ K40m GPU) using a two-step optimization procedure to minimize the loss function (Supplementary Material, Sect. 2). First, we use Adam’s algorithm^25^ for the first 1000 optimization steps (or epochs), with default learning rate 0.001 and parameters $\beta _1 = 0.9$, $\beta _2 = 0.999$. Then, we employ the limited memory BFGS, with bound constraints (L-BFGS-B) optimization algorithm^39,40^, implemented in the SciPy library^41^, with a maximum of $5 \times 10^4$ iterations. This training procedure is used by Lu *et al.* to train physics-informed NNs (PINNs) and mitigate the risk of the optimizer getting stuck at a poor local minima^42^. The L-BFGS-B algorithm is also successful for PDE learning^9^ and PDE solvers using DL techniques^37,42^. Moreover, this optimization algorithm takes advantage of the smoothness of the loss function by using second-order derivatives and often converges in fewer iterations than Adam’s algorithm and other methods based on stochastic gradient descent^42^. Within this setting, rational NNs are beneficial because the activation functions are smooth while maintaining an initialization close to ReLU (Supplementary Material, Fig. S2).

### Theoretical justification

Our approach for learning Green’s functions associated with linear differential operators has a theoretically rigorous underpinning. Indeed, it was shown in Ref.^17^ that uniformly elliptic operators in three dimensions have an intrinsic *learning rate*, which characterizes the number of training pairs needed to construct an $\epsilon$-approximation in the $L^2$-norm of the Green’s function, *G*, with high probability, for $0<\epsilon <1$. The number of training pairs depends on the quality of the covariance kernel used to generate the random forcing terms, $\{f_j\}_{j=1}^N$. Our choice of covariance kernel (Supplementary Material, Sect. 1) is motivated by the GP quality measure^17,43^, to ensure that our set of training forcing terms is sufficiently diverse to capture the action of the solution operator, $f\mapsto u(x) = \int _{\Omega }G(x,y)f(y)\,d y$, on a diverse set of functions.

Similarly, the choice of rational NNs to approximate the Green’s function, and the homogeneous solution, is justified by the higher approximation power of these networks over ReLU^19^. Other adaptive activation functions have been proposed for learning or solving PDEs with NNs^44^, but they are only motivated by empirical observations. Both theory and experiments support rational NNs for regression problems. The number of trainable parameters, consisting of weight matrices, bias vectors, and rational coefficients, needed by a rational NN to approximate smooth functions within $0<\epsilon <1$, can be completely characterized^19^. This motivates our choice of NN architecture for learning the Green’s functions.

## Supplementary information

Supplementary Information.

## Data Availability

All data and codes used in this article and the Supplementary Material are publicly available on the GitHub and Zenodo repositories at https://github.com/NBoulle/greenlearning/^45^ to reproduce the numerical experiments and figures. A software package, including additional examples and documentation, is also available at https://greenlearning.readthedocs.io/.

## References

[1] LeCun Y, Bengio Y, Hinton G (2015). Deep learning. Nature.

[2] Goodfellow I, Bengio Y, Courville A (2016). Deep Learning.

[3] Brunton SL, Noack BR, Koumoutsakos P (2020). Machine learning for fluid mechanics. Annu. Rev. Fluid Mech..

[4] Karniadakis GE (2021). Physics-informed machine learning. Nat. Rev. Phys..

[5] Brunton SL, Proctor JL, Kutz JN (2016). Discovering governing equations from data by sparse identification of nonlinear dynamical systems. Proc. Natl. Acad. Sci. U.S.A..

[6] Schaeffer H (2017). Learning partial differential equations via data discovery and sparse optimization. Proc. Math. Phys. Eng. Sci..

[7] Rudy SH, Brunton SL, Proctor JL, Kutz JN (2017). Data-driven discovery of partial differential equations. Sci. Adv..

[8] Zhang J, Ma W (2020). Data-driven discovery of governing equations for fluid dynamics based on molecular simulation. J. Fluid Mech..

[9] Raissi M (2018). Deep hidden physics models: Deep learning of nonlinear partial differential equations. J. Mach. Learn. Res..

[10] Lu L, Jin P, Pang G, Zhang Z, Karniadakis GE (2021). Learning nonlinear operators via DeepONet based on the universal approximation theorem of operators. Nat. Mach. Intell..

[11] Gin CR, Shea DE, Brunton SL, Kutz JN (2021). DeepGreen: Deep learning of Green’s functions for nonlinear boundary value problems. Sci. Rep..

[12] Li, Z., *et al.*, Neural operator: Graph kernel network for partial differential equations. Preprint at http://arxiv.org/abs/2003.03485 (2020).

[13] Feliu-Faba J, Fan Y, Ying L (2020). Meta-learning pseudo-differential operators with deep neural networks. J. Comput. Phys..

[14] Raissi M, Yazdani A, Karniadakis GE (2020). Hidden fluid mechanics: Learning velocity and pressure fields from flow visualizations. Science.

[15] Schmidt M, Lipson H (2009). Hidden fluid mechanics: Learning velocity and pressure fields from flow visualizations. Science.

[16] Udrescu M-L, Tegmark M (2020). AI Feynman: A physics-inspired method for symbolic regression. Sci. Adv..

[17] Boullé N, Townsend A (2022). Learning elliptic partial differential equations with randomized linear algebra. Found. Comput. Math..

[18] Stakgold I, Holst MJ (2011). Green’s Functions and Boundary Value Problems.

[19] Boullé N, Nakatsukasa Y, Townsend A (2020). Rational neural networks. Adv. Neural Inf. Process. Syst..

[20] Grüter M, Widman K-O (1982). The Green function for uniformly elliptic equations. Manuscr. Math..

[21] Dong H, Kim S (2009). Green’s matrices of second order elliptic systems with measurable coefficients in two dimensional domains. Trans. Am. Math. Soc..

[22] Kreyszig E (1978). Introductory Functional Analysis with Applications.

[23] Lee J-Y, Greengard L (1997). A fast adaptive numerical method for stiff two-point boundary value problems. SIAM J. Sci. Comput..

[24] Li, Z. *et al.* Fourier neural operator for parametric partial differential equations. In *International Conference on Learning Representations (ICLR)* (2021). PMC13460308 42583553

[25] Kingma, D. P. & Ba, J.: Adam: A method for stochastic optimization. In *International Conference on Learning Representations (ICLR)* (2015).

[26] Li, M., Zhang, T., Chen, Y. & Smola, A. J. Efficient mini-batch training for stochastic optimization. In *Proc. 20th ACM SIGKDD International Conference on Knowledge Discovery and Data Mining*, 661–670 (2014).

[27] Ascher UM, Ruuth SJ, Spiteri RJ (1997). Implicit-explicit Runge–Kutta methods for time-dependent partial differential equations. Appl. Numer. Math..

[28] Pareschi L, Russo G (2005). Implicit–explicit Runge–Kutta schemes and applications to hyperbolic systems with relaxation. J. Sci. Comput..

[29] Kelley CT (1995). Iterative Methods for Linear and Nonlinear Equations.

[30] Evans LC (2010). Partial Differential Equations.

[31] Arfken G, Weber H, Harris FE (2012). Mathematical Methods for Physicists.

[32] Myint-U T, Debnath L (2007). Linear Partial Differential Equations for Scientists and Engineers.

[33] Wight CL, Zhao J (2021). Solving Allen–Cahn and Cahn–Hilliard equations using the adaptive physics informed neural networks. Commun. Comput. Phys..

[34] Glorot, X., Bordes, A. & Bengio, Y. Deep Sparse Rectifier Neural Networks. *Proc. 14th International Conference on Artificial Intelligence and Statistics (AISTATS)* (2011), 315–323.

[35] Yarotsky D (2017). Error bounds for approximations with deep ReLU networks. Neural Netw..

[36] Glorot, X. & Bengio, Y. Understanding the difficulty of training deep feedforward neural networks. *Proc. 13th International Conference on Artificial Intelligence and Statistics*, 249–256 (2010).

[37] Raissi M, Perdikaris P, Karniadakis GE (2019). Physics-informed neural networks: A deep learning framework for solving forward and inverse problems involving nonlinear partial differential equations. J. Comput. Phys..

[38] Abadi, M. *et al.* TensorFlow: A System for Large-Scale Machine Learning. *12th USENIX Conference on Operating Systems Design and Implementation*, 265–283 (2016).

[39] Liu DC, Nocedal J (1989). On the limited memory BFGS method for large scale optimization. Math. Program..

[40] Byrd RH, Lu P, Nocedal J, Zhu C (1995). A limited memory algorithm for bound constrained optimization. SIAM J. Sci. Comput..

[41] Virtanen P (2020). SciPy 1.0: Fundamental algorithms for scientific computing in Python. Nat. Methods.

[42] Lu L, Meng X, Mao Z, Karniadakis GE (2021). DeepXDE: A deep learning library for solving differential equations. SIAM Rev..

[43] Boullé, N. & Townsend, A. A generalization of the randomized singular value decomposition.*International Conference on Learning Representations (ICLR)* (2022).

[44] Jagtap AD, Kawaguchi K, Karniadakis GE (2020). Adaptive activation functions accelerate convergence in deep and physics-informed neural networks. J. Comput. Phys..

[45] Boullé N (2021). NBoulle/GreenLearning—Software and datasets (version v10). Zenodo..

