# Supplementary material for: Data-driven discovery of Green’s functions with human-understandable deep learning
Source: Sci Rep. 2022 Mar 22;12:4824. doi: 10.1038/s41598-022-08745-5 (PMC8940897; doi:10.1038/s41598-022-08745-5)
Supplement: Supplementary file 1 — Supplementary Information. [file 41598_2022_8745_MOESM1_ESM.pdf]

Supplementary Material for

Data-driven discovery of Green's functions with human-understandable deep learning

Nicolas Boullé,* Christopher J. Earls, Alex Townsend

*Corresponding author. E-mail: boulle@maths.ox.ac.uk.

Contents

1 Generating the training data **3**

2 Loss function **5**

3 Measuring the results **7**

4 Robustness of the method **8**

4.1 Influence of the activation function on the accuracy 8

4.2 Number of training pairs and spatial measurements 10

4.3 Noise perturbation 11

4.4 Location of the measurements 12

4.5 Missing measurements data 12

5 Learning features of differential operators from the Green's function **13**

5.1 Linear constraints and symmetries 14

5.2 Eigenvalue decomposition 15

5.3 Singular value decomposition 18

5.4 Schrödinger equation with double-well potential 20

5.5 Singularity location and type 21

6 Differential operators in two dimensions 23

7 System of differential equations 26

8 Analysis of main text examples 27

8.1 Viscous shock 28

8.2 Advection-diffusion on the right of the domain 28

8.3 Linearized models of nonlinear operators 28

8.4 Lid-driven cavity problem 29

9 Time-dependent equations 33

1 Generating the training data

The training dataset consists of N forcing functions, $f_j : \Omega \to \mathbb{R}$, and associated system responses, $u_j : \Omega \to \mathbb{R}$, which are solutions to the following equation:

$$\mathcal{L}u_j = f_j, \qquad \mathcal{D}(u_j, \Omega) = g, \tag{1}$$

where $\mathcal{L}$ is a linear differential operator, $\mathcal{D}$ is a linear operator acting on the solutions, uj, and the domain, Ω; with g being the constraint. Unless otherwise stated, the training data comprises $N = 100$ forcing and solution pairs, where the forcing terms are drawn at random from a Gaussian process, $\mathcal{GP}(0, K_{\text{SE}})$, where K_{SE} is the squared-exponential covariance kernel [1] defined as

$$K_{\text{SE}}(x, y) = \exp\left(-\frac{|x-y|^2}{2\ell^2}\right), \qquad x, y \in \Omega. \tag{2}$$

The parameter $\ell > 0$ in Eq. (2) is called the length-scale parameter, and characterizes the correlation between the values of $f \sim \mathcal{GP}(0, K_{\text{SE}})$ at x and y for $x, y \in \Omega$. A small parameter, ℓ, yields highly oscillatory random functions, f, and determines the ability of the GP to generate a diverse set of training functions. This last property is crucial for capturing different modes within the operator, $\mathcal{L}$, and for learning the associated Green's function accurately [2]. Other possible choices of covariance kernels include the periodic kernel [1]:

$$K_{\text{Per}}(x, y) = \exp\left(-\frac{2\sin^2(\pi|x-y|)}{\ell^2}\right), \qquad x, y \in \Omega,$$

which is used to sample periodic random functions for problems with periodic boundary conditions (Fig. S6B). Another possibility is a kernel from the Matérn family [1].

When Ω is an interval $[a, b]$, we introduce a normalized length-scale parameter $\lambda = \ell/(b-a)$, so that the method described does not depend on the length of the interval. In addition, we choose $\lambda = 0.03$, so that the length-scale, ℓ, is larger than the forcing spatial discretization size, which allows us to adequately resolve the functions sampled from the GP with the discretization.

More precisely, we make sure that $\ell \geq (b-a)/N_f$ so that $1/N_f \leq \lambda$. In Fig. S1, we display the squared-exponential covariance kernel on the domain $\Omega = [-1, 1]$, along with ten random functions sampled from $\mathcal{GP}(0, K_{\text{SE}})$.

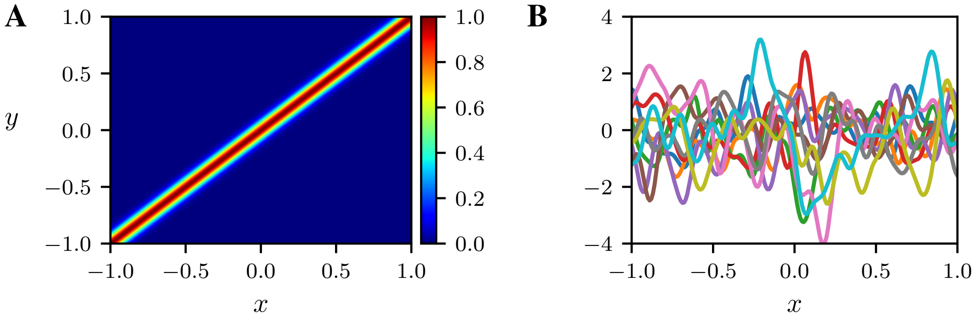

Figure S1: Random forcing terms. Squared exponential covariance kernel K_{SE} on $[-1, 1]^2$ with normalized length-scale $\lambda = 0.03$ (A) together with 10 functions sampled from the Gaussian process $\mathcal{GP}(0, K_{\text{SE}})$ (B).

When Eq. (1) is a boundary-value problem, we generate training pairs by solving the PDE with a spectral method [3] using the Chebfun software system [4], written in MATLAB, and using a tolerance of 5×10^{-13}. We also solve the homogeneous problem with zero-forcing, to compare the learned and exact homogeneous solutions. The exact homogeneous solution is not included in the training dataset. When the homogeneous solution is zero, the solutions, $\{u_j\}_{j=1}^N$, and forcing terms, $\{f_j\}_{j=1}^N$, are rescaled, so that $\max_{1\leq j\leq N} \|u_j\|_{L^\infty(\Omega)} = 1$. By doing this, we facilitate the training of the NNs by avoiding disproportionately small-scale or large-scale data. In the presence of real data, with no known homogeneous solution, one could instead normalize the output of the NNs, $\mathcal{N}_G$ and $\mathcal{N}_{\text{hom}}$, to facilitate the training procedure.

2 Loss function

The NNs, $\mathcal{N}_G$ and $\mathcal{N}_{\text{hom}}$, are trained by minimizing a mean square relative error (in the L^2-norm) regression loss, defined as:

$$\text{Loss} = \frac{1}{N}\sum_{j=1}^{N}\frac{1}{\|u_j\|^2_{L^2(\Omega)}}\int_\Omega\left(u_j(x)-\mathcal{N}_{\text{hom}}(x)-\int_\Omega\mathcal{N}_G(x,y)f_j(y)\,\mathrm{d}y\right)^2\mathrm{d}x. \tag{3}$$

Unless otherwise stated, the integrals in Eq. (3) are discretized by a trapezoidal rule [5] using training data values that coincide with the forcing discretization grid, $\{y_i\}_{i=1}^{N_f}$, and measurement points, $\{x_i\}_{i=1}^{N_u}$. As an example, for $1 \leq j \leq N$, the squared L^2-norm of u_j, on a one-dimensional domain $\Omega = [a,b] \subset \mathbb{R}$, is approximated as

$$\|u_j\|^2_{L^2(\Omega)} = \int_a^b u_j(x)^2\,\mathrm{d}x \approx \sum_{i=2}^{N_u}\frac{u_j(x_{i-1})^2+u_j(x_i)^2}{2}\Delta_{x_i},$$

where $\Delta_{x_i} = x_i - x_{i-1}$ is the length of the ith subinterval $[x_{i-1}, x_i]$.

In Section 4.4, we compare the results obtained by using trapezoidal integration, described above, and a Monte-Carlo integration [6]:

$$\|u_j\|^2_{L^2(\Omega)} \approx \frac{b-a}{N_u}\sum_{i=1}^{N_u}u_j(x_i)^2,$$

which has a lower convergence rate to the integral with respect to the number of points, N_u. This integration technique is, however, particularly suited for approximating integrals in high dimensions, or with complex geometries [6].

It is also possible to enforce some prior knowledge, regarding the Green's function, through the loss function, by adding a penalty term. If the differential operator is self-adjoint, then depending on the constraint operator $\mathcal{D}$, the associated Green's function is symmetric, *i.e.*, $G(x,y) = G(y,x)$ for all $x, y \in \Omega$. In this case, one can train a symmetric NN $\mathcal{N}_G$ defined as

$$\mathcal{N}_G(x,y) = \mathcal{N}(x,y) + \mathcal{N}(y,x), \qquad x,y \in \Omega.$$

However, our numerical experiments reveal that the NNs can learn both boundary conditions and symmetry properties directly, from the training data, without additional constraints on the loss function or network architectures.

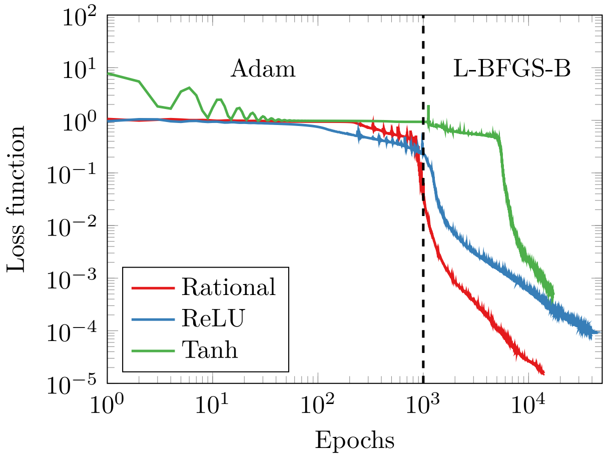

Figure S2: Loss function during training. Loss function magnitudes for the ReLU, tanh, and rational NNs with respect to the number of epochs. The networks are trained to learn the Green's function of the Helmholtz operator with homogeneous Dirichlet boundary conditions and frequency $K = 15$. Adam's optimizer is used until 1000 epochs (before the dashed line) and L-BFGS-B is employed thereafter.

In Fig. S2, we display the value of the loss function during the training of the NNs with different activation functions: rational, ReLU, and hyperbolic tangent (tanh). In this example, we aim to learn the Green's function of a high-frequency Helmholtz operator with homogeneous Dirichlet boundary conditions on $\Omega = [0, 1]$:

$$\mathcal{L}u = \frac{d^2u}{dx^2} + K^2u, \qquad u(0) = u(1) = 0, \tag{4}$$

where $K = 15$ denotes the Helmholtz frequency. We first remark in Fig. S2 that the rational NN is easier to train than the other NNs, as it minimizes the loss function to 10^{-5} with ≈ 15000 epochs, while a ReLU NN requires three times as many optimization steps to reach 10^{-4}. We also see that the loss function for the ReLU and rational NN becomes more oscillatory [7] and harder to minimize before epoch 1000, while it converges much faster after switching to

L-BFGS-B. In theory, one could introduce a variable learning rate that improves the behavior of Adam's optimizer [8, 9]. However, that introduces an additional parameter, which is not desirable in the context of PDE learning. We aim to design an adaptive and easy-to-use method that does not require extensive hyperparameter tuning. We also observe that the tanh NN has a similar convergence rate to the rational NN due to the smoothness of the activation function, but this network exhibits instability during training, as indicated by the high value of the loss function when the optimization terminates. Rational NNs do not suffer from this issue, thanks to the initialization close to a ReLU NN, as can be observed in Fig. S2, when focusing on the value of the loss function corresponding to the early optimization steps.

3 Measuring the results

Once the NNs have been trained, we visualize the Green's functions by sampling the networks on a fine 1000×1000 grid of $\Omega \times \Omega$. In the case where the exact Green's function G_{exact} is known, we measure the accuracy of the trained NN, $\mathcal{N}_G$, using a relative error in the L^2-norm:

$$\text{Relative Error} = 100 \times \|G_{\text{exact}} - \mathcal{N}_G\|_{L^2(\Omega)} / \|G_{\text{exact}}\|_{L^2(\Omega)}. \tag{5}$$

Here, we multiplied by 100 to obtain the relative error as a percentage (%). This illustrates an additional advantage of using a Green's function formulation: we can create test case problems with known Green's functions and evaluate the method using relative error and offer performance guarantees. The standard approaches in the literature often use best-case and worst-case examples as testing procedures and therefore do not guarantee that the solution operator is accurately learned. The "worst-case" examples can be misleading if they consist of functions with similar behavior to the forcing terms already included in the training dataset. Furthermore, since the space of possible forcing terms is of infinite dimension, it is not possible to evaluate the trained NNs on all these functions to obtain a true worst-case example.

4 Robustness of the method

We test the robustness of our DL method for learning Green's functions and homogeneous solutions of differential equations, with respect to the number of training pairs, the discretization of the solutions and forcing terms, and the noise perturbation of the training solutions, $\{u_j\}_{j=1}^N$. For consistency, we perform numerical experiments where we learn the Green's function of the Helmholtz operator with parameter $K = 15$ and homogeneous Dirichlet boundary conditions (see Eq. (4)). The performance is measured using the relative error in the L^2-norm defined in Eq. (5) between the trained network, $\mathcal{N}_G$, and the exact Green's function, G_{exact}, whose analytic expression is given by

$$G_{\text{exact}}(x, y) = \begin{cases} \frac{\sin(15x)\sin(15(y-1))}{15\sin(15)}, & \text{if } x \leq y, \\ \frac{\sin(15y)\sin(15(x-1))}{15\sin(15)}, & \text{if } x > y, \end{cases}$$

where $x, y \in [0, 1]$.

4.1 Influence of the activation function on the accuracy

We compare the performances of different activation functions for learning the Green's functions of the Helmholtz operator by training the NNs, $\mathcal{N}_G$ and $\mathcal{N}_{\text{hom}}$, with rational, ReLU, and tanh activation functions. The numerical experiments are repeated ten times to study the statistical effect of the random initialization of the network weights and the stochastic nature of Adam's optimizer. The rational NN achieves a mean relative error of 1.2% (with a standard deviation of 0.2%), while the ReLU NN reaches an average error of 3.3% (with a standard deviation of 0.2%), which is three times larger. Note that the ten times difference in the loss function between ReLU and Rational NNs, displayed in Fig. S2, is consistent with the factor of three in the relative error since the loss is a mean squared error and $\sqrt{10} \approx 3$. This indicates that the rational neural networks are not overfitting the training dataset. One of the numerical experiments with a tanh NN terminated early due to the training instabilities mentioned in Section 2:

achieving a relative error of 99%. We excluded this problematic run when comparing the ReLU and rational NN's accuracy, limiting ourselves only to cases where the training was successful. The ReLU and rational NNs did not suffer from such issues and were always successful. The averaged relative error of the tanh NN, over the nine remaining experiments, is equal to 3.9% (with a standard deviation of 1.4%), which is slightly worse than the ReLU NN, with higher volatility of the results.

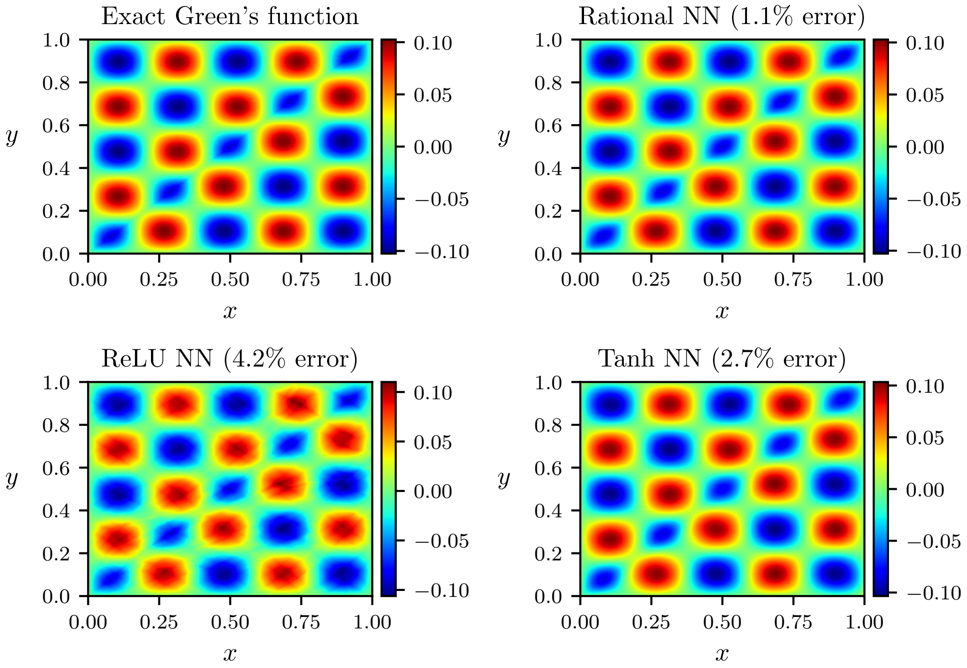

Figure S3: Comparison of activation functions. Exact and learned Green's functions of the Helmholtz operator by a rational, ReLU, and tanh NN. The relative error in the L^2 norm is reported in the titles of the panels.

The exact and learned Green's functions with rational, ReLU, and tanh NNs are displayed in Fig. S3. We see that the rational and tanh NNs are smooth approximations of the exact Green's function, while visual artifacts are present for the ReLU NN as it is piecewise linear, despite its good accuracy.

4.2 Number of training pairs and spatial measurements

This section describes our method's accuracy as we change the number of training pairs and the size of the spatial discretization. First, we fix the number of spatial measurements to be $N_u = 100$, and then vary the number of input-output pairs, $\{(f_j, u_j)\}_{j=1}^N$, of the training dataset for the Helmholtz operator with Dirichlet boundary conditions (see Eq. (4)). As we increase N from 1 to 100, we report the relative error of the Green's function learned by a rational NN in Fig. S4A. Next, in Fig. S4B, we display the relative error on the learned Green's function as we increase N_u from 3 to 100, with $N = 100$ input-output pairs. Note that we only perform the numerical experiments once since we obtained a low variation of the relative errors in Section 4.1 when the networks, $\mathcal{N}_G$ and $\mathcal{N}_{\text{hom}}$, have rational activation functions. We observe similar behavior in Fig. S4A and B, where the relative error first rapidly (exponentially) decreases as we increase the number of functions in our dataset or spatial measurements of the solutions to the Helmholtz equations with random forcing terms. One important thing to notice is our method's ability to learn the Green's function of a high-frequency Helmholtz operator, with only 1% relative error, using very few training pairs. The performance reaches a plateau at $N \approx 20$ and $N_u \approx 20$, respectively, and ceases to improve. However, the stagnation of the relative error for more numerous training data and spatial measurements is expected and can be explained by our choice of covariance kernel length-scale, which restricts the GP's ability to generate a wide variety of forcing terms. This issue can be resolved by decreasing the length-scale parameter and concomitantly increasing the forcing discretization size or choosing another covariance kernel with a less pronounced eigenvalue decay rate [2].

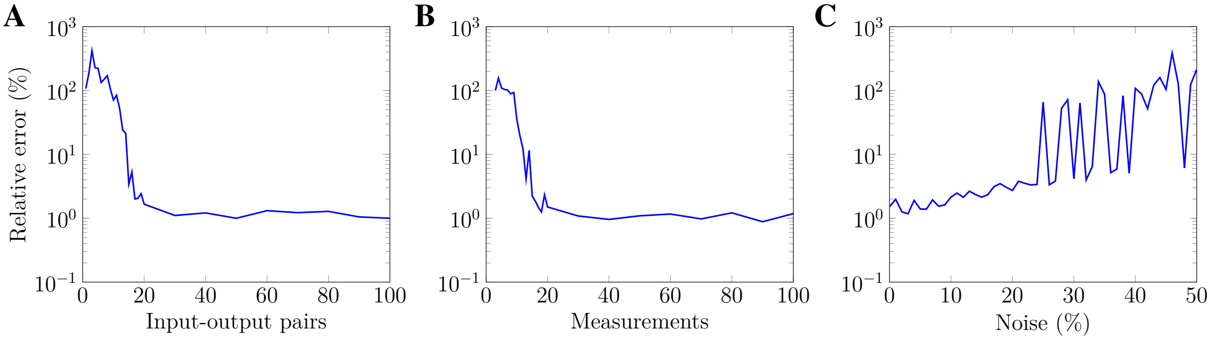

Figure S4: Robustness of the method. Relative error of the learned Green's function of the Helmholtz operator with respect to the number of input-output pairs (A), spatial measurements (B), and level of Gaussian noise perturbation (C).

4.3 Noise perturbation

The impact of noise in the training dataset on the accuracy of the learned Green's function is gauged by perturbing the system's response measurements with Gaussian noise as

$$u_j^{\text{noise}}(x_i) = u_j(x_i)(1 + \delta c_{i,j}), \tag{6}$$

where the coefficients $c_{i,j}$ are independent and identically distributed, Gaussian random variables for $1 \leq i \leq N_u$ and $1 \leq j \leq N$, and δ denotes the noise level (in percent). We then vary the level of Gaussian noise perturbation from 0% to 50%, train the NNs, N_G and N_{hom}, for each choice of the noise level, and report the relative error in Fig. S4C. We first observe a low impact of the noise level on the accuracy of the learned Green's function, as a perturbation of the system's responses measurements with 20% noise only increases the relative error by a factor of 2 from 1.5% (no noise) to 2.7%. When the level of noise exceeds 25%, we notice large variations of the relative errors and associated higher volatility in results, characterized by a large standard deviation in error associated with repeated numerical experiments. We consider our DL approach relatively robust to noise in the training dataset.

4.4 Location of the measurements

As described in the *Methods* and Section 1, by default, we use a uniform grid for spatial measurements of the training dataset, and thus we discretize the integrals in the loss function (cf. Eq. (3)) using a trapezoidal rule. We conducted additional numerical experiments on the Helmholtz example to study the influence of the measurements' location and quadrature rule on the relative error of the learned Green's function. We report the relative errors between the learned and exact Green's functions in Table S1, using a Monte-Carlo or a trapezoidal rule to approximate the integrals and uniform or random spatial measurements. In the latter case, the measurement points $\{x_i\}_{i=1}^{N_u}$ are independently and identically sampled from a uniform distribution, $\mathcal{U}(0,1)$, where $\Omega = [0,1]$ is the domain. We find that the respective relative errors vary between 0.96% and 1.3%. Therefore, we do not observe statistically significant differences in the relative error computed by rational NNs. These results support the claim that our method is relatively robust to the type of spatial measurements in the training dataset.

Table S1: Choice of quadrature rules. Relative error of the Green's function of the Helmholtz operator with frequency $K = 15$ learned by a rational NN with respect to the type of spatial measurements and quadrature rule (Monte-Carlo or trapezoidal rule) used.

Spatial measurements	Monte-Carlo	Trapezoidal rule
Random	1.1%	1.3%
Uniform	1.3%	0.96%

4.5 Missing measurements data

Since experimental data may be partially corrupted or unavailable at some spatial locations, we assess our method's accuracy with respect to missing measurement data in the training dataset. We consider the high-frequency Helmholtz operator, defined on the domain $\Omega = [0,1]$ by Eq. (4), with homogeneous Dirichlet boundary conditions. We introduce a gap in the spatial measurements located at $x \in [0.5, 0.7]$ by sampling the system's responses, $\{u_j\}_{j=1}^{N}$, uniformly

on the domain, $[0, 0.5] \cup [0.7, 1]$. Note that the forcing terms, $\{f_j\}_{j=1}^N$, are still sampled uniformly on the whole domain since they are assumed to be known. The Green's function and homogeneous solution learned by the rational NNs are displayed in Fig. S5A and B, respectively. Surprisingly, we find that the NN, $\mathcal{N}_G$, can capture the high-frequency pattern of the Green's function and achieves a relative error of 8.2%, despite the large gap within the measurement data for $x \in [0.5, 0.7]$. Another interesting outcome of this numerical experiment is that the lack of spatial measurements in a specific interval does not influence the accuracy of our method outside this location, *i.e.*, for $x \in [0, 0.5] \cup [0.7, 1]$ and $y \in [0, 1]$ in this example.

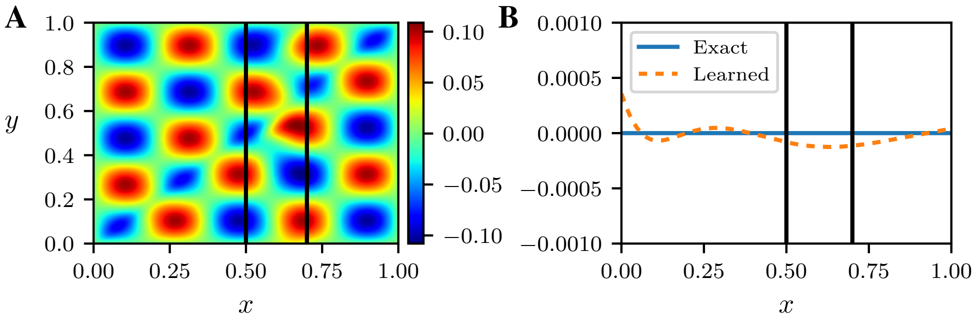

Figure S5: Gap in measurements. (A) Green's function of the Helmholtz operator and its homogeneous solution (B) learned by a rational NN with no measurement points for $x \in [0.5, 0.7]$. The space between the vertical black lines indicates where there is a lack of spatial measurements.

5 Learning features of differential operators from the Green's function

This section highlights that several features of the differential operators can be extracted from the learned Green's function, which supports our aim of uncovering mechanistic understanding from input-output pairs of forcing terms and solutions.

5.1 Linear constraints and symmetries

We first remark that boundary constraints, such as the constraint operator, $\mathcal{D}$, of Eq. (1), can be recovered from the Green's function, G, of the differential operator, $\mathcal{L}$. Hence, let $f \in C_c^\infty(\Omega)$ be any infinitely differentiable function with a compact support on Ω, and u be the solution to Eq. (1) with forcing term, f, such that

$$u(x) = \int_\Omega G(x,y) f(y)\,\mathrm{d}y + u_{\text{hom}}(x), \qquad x \in \Omega.$$

Under sufficient regularity conditions, the linearity of the operator, $\mathcal{D}$, implies that $\mathcal{D}(G(\cdot,y),\Omega) = 0$ for all $y \in \Omega$. For instance, if $\mathcal{D}$ is the Dirichlet operator: $\mathcal{D}(u,\Omega) = u_{|\partial\Omega}$, then the Green's function satisfies $G(x,y) = 0$ for all $x \in \partial\Omega$.

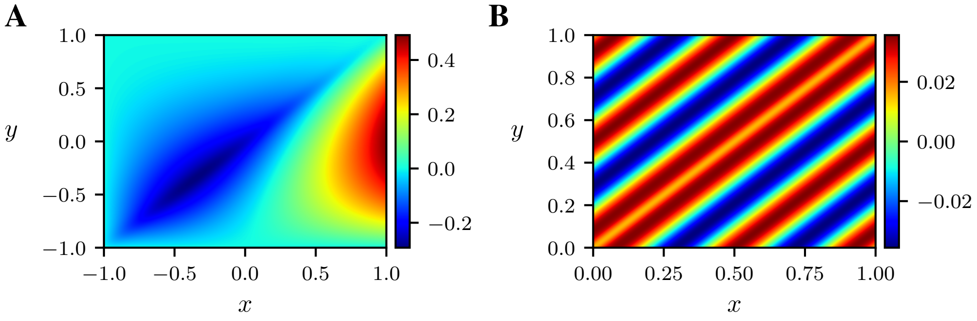

Figure S6: Extraction of linear constraints. (A) Learned Green's functions of a second-order differential operator with an integral constraint defined in Eq. (7). (B) Green's function of the Helmholtz operator with periodic boundary conditions learned by a rational NN.

As an example, we display in Fig. S6A the learned Green's function of the following second-order differential operator on $\Omega = [-1, 1]$ with an integral constraint on the solution:

$$\mathcal{L}u = \frac{du^2}{dx^2} + x^2 u, \qquad u(-1) = 1, \qquad \int_{-1}^{1} u(x)\,\mathrm{d}x = 2. \tag{7}$$

We observe that $G(-1,y) = 0$ for all $y \in [-1,1]$ and one can verify that $\int_{-1}^{1} G(x,y)\,\mathrm{d}x = 0$ for any $y \in [-1,1]$. In a second example, we learn the Green's function of the Helmholtz operator on $\Omega = [0,1]$ with frequency $K = 15$ and periodic boundary conditions: $u(0) = u(1)$.

One can see in Fig. S6B that the Green's function itself is periodic and that $G(0, y) = G(1, y)$ for all $y \in [0, 1]$, as expected. The periodicity of the Green's function in the y-direction: $G(x, 0) = G(x, 1)$ for $x \in [0, 1]$, is due to the fact that the Helmholtz operator is self-adjoint, which implies symmetry in the associated Green's function. Furthermore, any linear constraint $\mathcal{C}(u) = 0$ such as linear conservation laws or symmetries [10], satisfied by all the solutions to Eq. (1), under forcing $f \in C_c^\infty(\Omega)$, is also satisfied by the Green's function, G, such that $\mathcal{C}(G(\cdot, y)) = 0$ for all $y \in \Omega$, and is therefore witnessed by the Green's function.

5.2 Eigenvalue decomposition

Let $\mathcal{L}$ be a self-adjoint operator and consider the following eigenvalue problem:

$$\mathcal{L}v = \lambda v, \qquad \mathcal{D}(v, \Omega) = 0, \tag{8}$$

where v is an eigenfunction of the differential operator, $\mathcal{L}$, satisfying the homogeneous constraints with associated eigenvalue, $\lambda > 0$. The eigenfunction, v, can be expressed using the Green's function, G, of $\mathcal{L}$ as

$$v(x) = \lambda \int_\Omega G(x, y) v(y) \, \mathrm{d}y, \qquad x \in \Omega,$$

which implies that v is also an eigenfunction of the integral operator with kernel G, but with eigenvalue $1/\lambda$. Consider now the eigenvalue problem associated with the Green's function, itself:

$$\int_\Omega G(x, y) w(y) \, \mathrm{d}y = \mu w(x), \qquad x \in \Omega,$$

where $\mu > 0$. Then, we find that $(w, 1/\mu)$ are solutions to the eigenvalue problem (8). Consequently, the differential operator, $\mathcal{L}$, and integral operator with kernel, G, share the same eigenfunction, but possess reciprocal eigenvalues [11]. Thus, we can effectively compute the lowest eigenmodes of $\mathcal{L}$ from the learned Green's function.

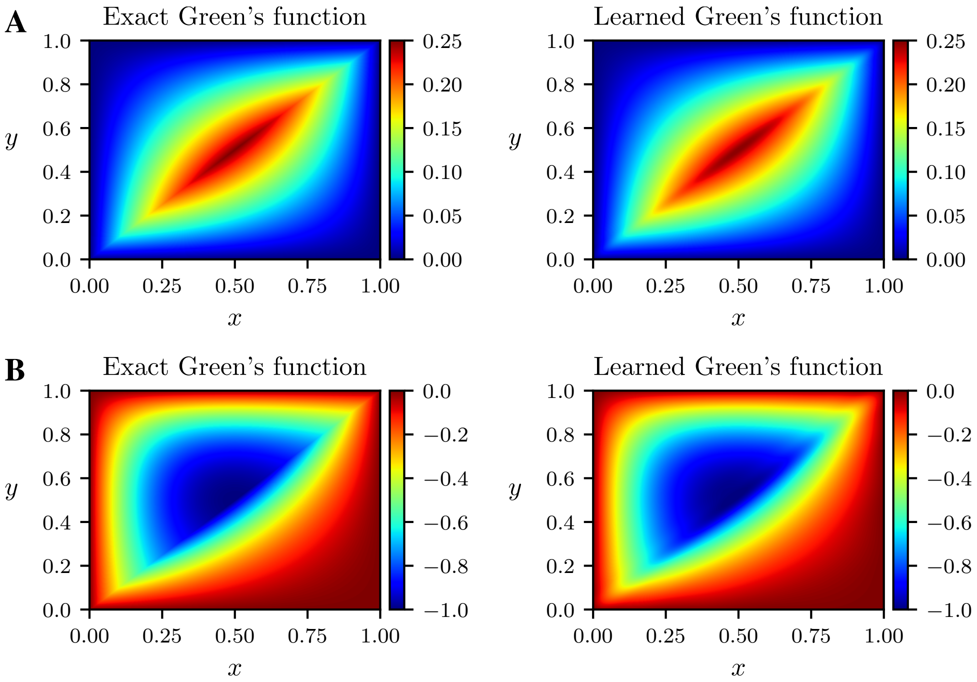

Figure S7: Laplace and advection-diffusion operators. Exact and learned Green's functions of the Laplace (A) and advection-diffusion (B) operators.

We now evaluate our method's ability to accurately recover the eigenfunctions of the Green's function that are associated with the largest eigenvalues, in magnitude, from input-output pairs. We train a NN to learn the Green's function of the Laplace operator $\mathcal{L}u = -d^2u/dx^2$ on $[0, 1]$, with homogeneous Dirichlet boundary conditions, and numerically compute its eigenvalue decomposition. In Fig. S7A, we display the learned and exact Green's function, whose expression is given by

$$G_{\text{exact}}(x, y) = \begin{cases} x(1-y), & \text{if } x \leq y, \\ y(1-x), & \text{if } y < x, \end{cases}$$

for $x, y \in [0, 1]$.

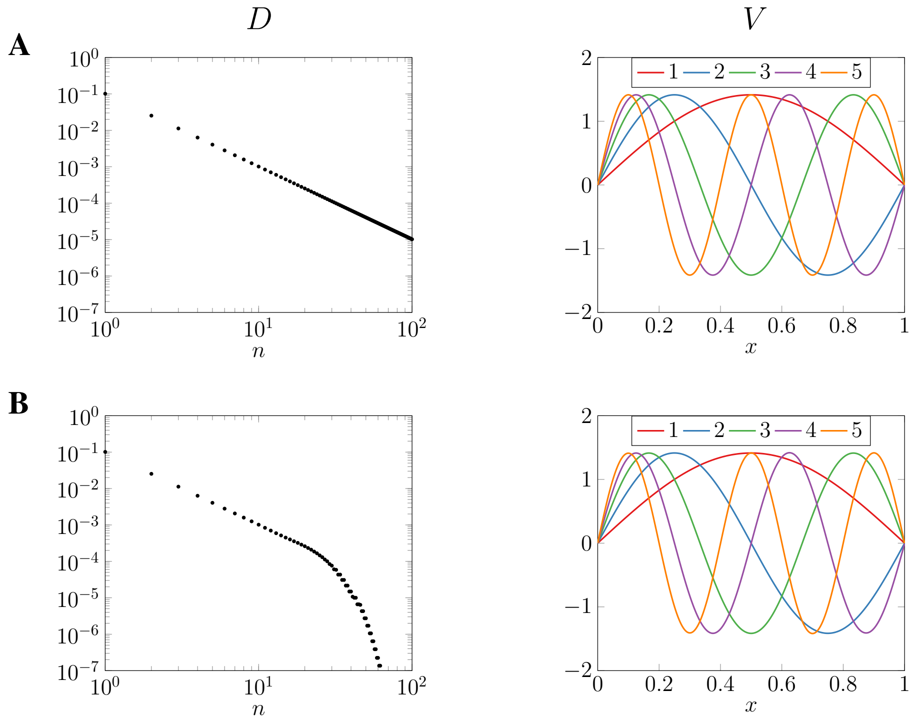

Figure S8: Eigenvalue decomposition. The first 100 largest eigenvalues and first five eigenfunctions of the exact (A) and learned (B) Green's functions of the Laplace operator. The eigenvalues are represented in the left panels, while the right panels illustrate the first five eigenfunctions of the Green's function.

The one hundred largest eigenvalues in magnitude, along with the corresponding first five eigenfunctions, are visualized for the exact and learned Green's functions in Fig. S8. Note

that the eigenvectors of the learned Green's functions are normalized and flipped to match the ones of the exact Green's function because eigenfunctions are unique up to a scalar multiple when the eigenvalues are all distinct. We find that we can recover the largest eigenvalues and eigenfunctions of the learned Green's function and that the first 20 largest eigenvalues remain accurate. Therefore, the approximation error between the learned and exact Green's functions mainly affects the smallest eigenvalues. This is an essential feature of our method since the dominant eigenmodes of the differential operator $\mathcal{L}$ are associated with the largest eigenvalues of the Green's functions, which can be learned accurately. The exponential decay of the smallest eigenvalues of the learned Green's function in the left panel of Fig. S8B is because the rational NN is a smooth approximation to the exact Green's function.

5.3 Singular value decomposition

When the Green's function of the differentiation operator, $\mathcal{L}$, is square-integrable, its associated Hilbert–Schmidt integral operator defined by

$$\mathscr{F}_G f(x) = \int_\Omega G(x,y)f(y)\,\mathrm{d}y, \qquad x \in \Omega,$$

is compact and admits a singular value decomposition (SVD) [12]. Then, there exist a positive sequence $\sigma_1 \geq \sigma_2 \geq \cdots > 0$, and two orthonormal bases, $\{\phi_n\}$ and $\{\psi_n\}$, of $L^2(\Omega)$ such that

$$u(x) = \int_\Omega G(x,y)f(y)\,\mathrm{d}y + u_{\text{hom}}(x) = \sum_{n=1}^{\infty} \sigma_n \langle \phi_n, f\rangle \psi_n(x) + u_{\text{hom}}(x), \qquad x \in \Omega, \tag{9}$$

where u is the solution to Eq. (1) with forcing term f, and $\langle \cdot, \cdot \rangle$ denotes the inner product in $L^2(\Omega)$. Therefore, the action of the solution operator $f \mapsto u$ can be approximated using the SVD of the integral operator. Similarly to Section 5.2 with the eigenvalue decomposition, the dominant terms in the expansion of Eq. (9) are associated with the largest singular values $\sigma_1 \geq \sigma_2 \geq \cdots > 0$ of the integral operator.

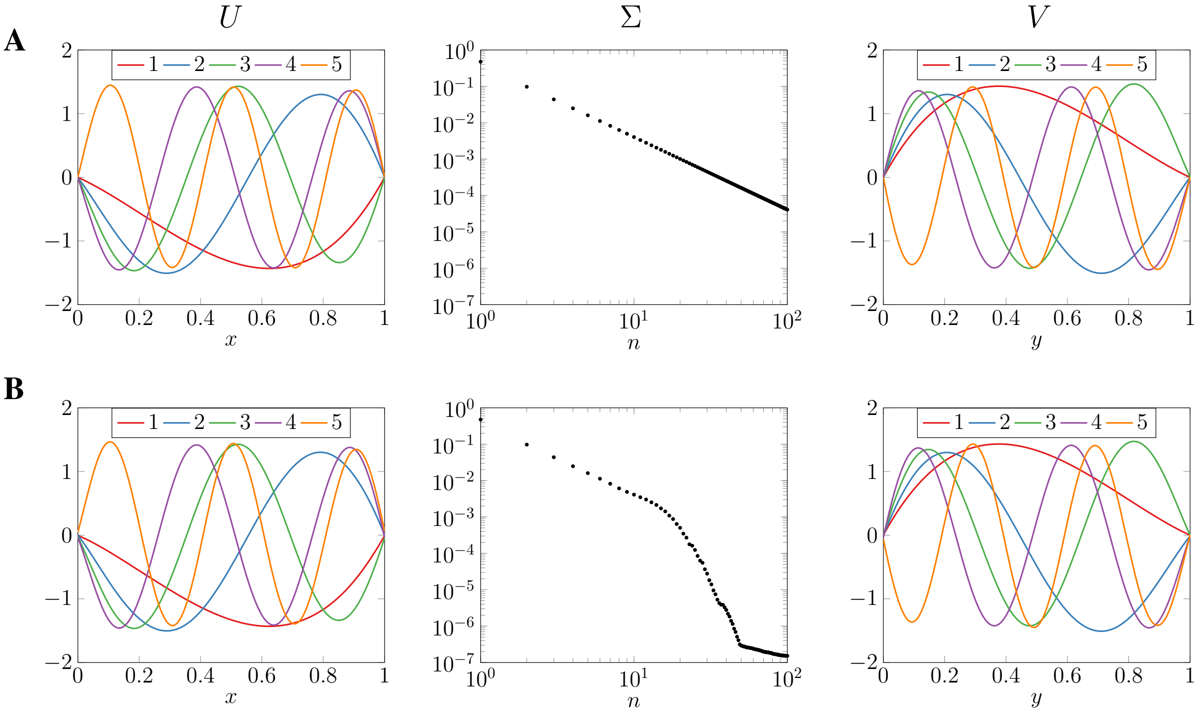

Figure S9: Singular value decomposition. Singular value decomposition of the exact (A) and learned (B) Green's functions of the advection-diffusion operator defined by Eq. (10). The left and right panels, respectively, show the first five left and right singular vectors, $\{\phi\}_{n=1}^{5}$ and $\{\psi\}_{n=1}^{5}$, of the exact and learned Green's functions. The singular values of the Green's functions are plotted in the middle panel.

We now show that one can accurately recover the first singular values and singular vectors from the Green's function learned by a rational NN. We train a rational NN to learn the Green's function of an advection-diffusion operator $\mathcal{L}$ on $\Omega = [0,1]$ with Dirichlet boundary conditions, defined as

$$\mathcal{L}u = \frac{1}{4}\frac{d^2u}{dx^2} + \frac{du}{dx} + u, \qquad u(0) = 1,\ u(1) = -2. \tag{10}$$

The learned Green's function is illustrated in Fig. S7A, next to the exact Green's function given by:

$$G_{\text{exact}}(x,y) = \begin{cases} 4x(y-1)\exp(-2(x-y)), & \text{if } x \leq y, \\ (x-1)y, & \text{if } y < x, \end{cases}$$

for $x, y \in [0,1]$. In Fig. S9, we display the first five left and right singular vectors and the singular values of the exact and learned Green's functions. We observe that the first fifteen singular values of the learned Green's functions are accurate. This leads us to conclude that our method enables the construction of a low-rank representation of the solution operator associated with the differential operator, $\mathcal{L}$, and allows us to compute and analyze its dominant modes.

5.4 Schrödinger equation with double-well potential

We highlight the ability of our DL method to learn physical features of an underlying system by considering the steady-state one-dimensional Schrödinger operator on $\Omega = [-3,3]$:

$$\mathcal{L}(u) = -h^2\frac{d^2u}{dx^2} + V(x)u, \qquad u(-3) = u(3) = 0,$$

with double-well potential $V(x) = x^2 + 1.5\exp(-(4x)^4)$ and $h = 0.1$ [13]. The potential $V(x)$ is illustrated in Fig. S10, along with the Green's function learned by the rational NN from pairs of forcing terms and the system's responses. First, the shape of the well potential can be visualized along the diagonal of the Green's function in Fig. S10B. Next, in Fig. S10, we compute the first ten eigenstates of the Schrödinger operator in Chebfun [4] and plot them using a similar representation as Fig. 6.9 of [13]. Similarly to Section 5.2, we compute the eigenvalue

decomposition of the Green's function learned by a rational NN and plot the eigenstates (shifted by the corresponding eigenvalues) in Fig. S10. Note that the eigenvalues of the operator and the Green's functions are reversed. We observe a perfect agreement between the first ten exact and learned eigenstates. These energy levels capture information about the states of atomic particles modeled by the Schrödinger equation.

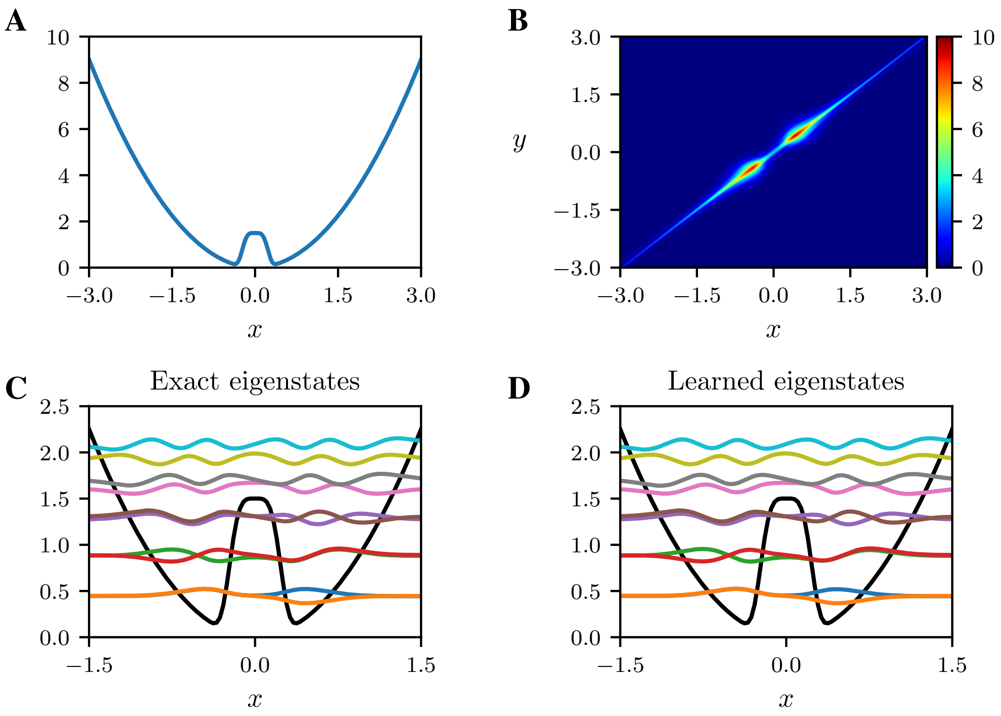

Figure S10: Schrödinger equation. (A) Double well potential $V(x) = x^2 + 1.5\exp(-(4x)^4)$. (B) Learned Green's function of the Schrödinger equation with potential $V(x)$. (C) First ten exact eigenstates computed numerically from the Schrödinger operator and (D) eigenstates computed from the learned Green's function displayed in B. The eigenfunctions are shifted by an amount corresponding to the eigenvalue. The double-well potential is shown as a black curve.

5.5 Singularity location and type

The input-output function of a rational NN is a high-degree rational function, which means that it has poles (isolated points for which it is infinite). In rational function approximation theory, it

is known that the poles of a near-optimal rational approximant tend to cluster near a function's singularities [14]. The clustering of the poles near the singularity is needed for the rational approximant to have excellent global approximation [15, 16]. Moreover, the type of clustering (algebraic, exponential, beveled exponential) can reveal the type of singularity (square-root, blow-up, non-differentiable) at that location. This feature of rational approximants is used in other settings [17].

We show that the rational NNs also cluster poles in a way that identifies its location and type. In Fig. S11C, we display the complex argument of the trained rational NN for the Green's function of a second-order differential operator with a jump condition, defined on $\Omega = [0, 1]$ as

$$\mathcal{L}u = 0.2\frac{d^2u}{dx^2} + \frac{du}{dx}, \qquad u(0) = u(1) = 0,\, u(0.7^-) = 2,\, u(0.7^+) = 1.$$

These diagrams are known as phase portraits and are useful for illustrating complex analysis [18]. A pole of the rational function can be identified as a point in the complex plane for which the full colormap goes around that point in a clockwise fashion. In particular, in Fig. S11C, we see that the poles of the rational function cluster quite closely to the real-line (where $Im(z) = 0$) at $x = 0.7$. If the clustering is examined more closely, it may be possible to reveal that the singularity in the Green's function at $x = 0.7$ is due to a jump condition.

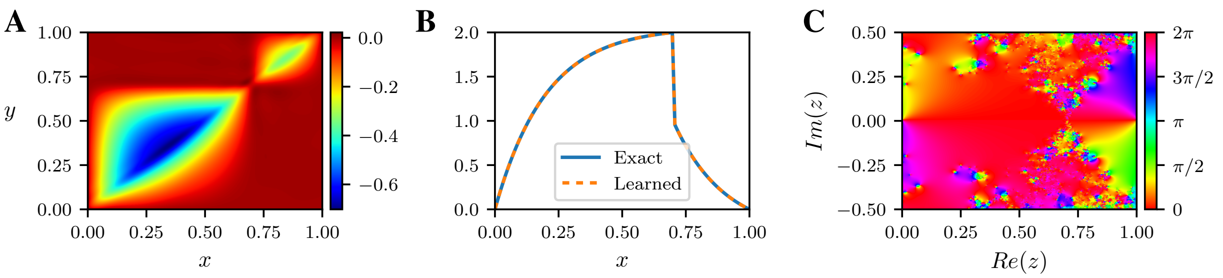

Figure S11: Singularity location. (A) Learned Green's function of a second-order differential operator with a jump condition at $x = 0.7$. Homogeneous solution of the operator with jump condition (B) and argument of the rational NN representing the homogeneous solution in the complex plane (C).

Rational NNs are also important for resolving Green's function with boundary layers as the NN can resolve the boundary layer by clustering its poles in the complex plane. In Fig. S12, we see a learned Green's function of a differential equation with a boundary layer at $x = 0$:

$$\mathcal{L}u = -10^{-2}\frac{d^2u}{dx^2} - \frac{du}{dx}, \qquad u(0) = u(1) = 0, \qquad \Omega = [0, 1].$$

While the Green's function is not smooth, our rational NN still resolves it with relatively good accuracy.

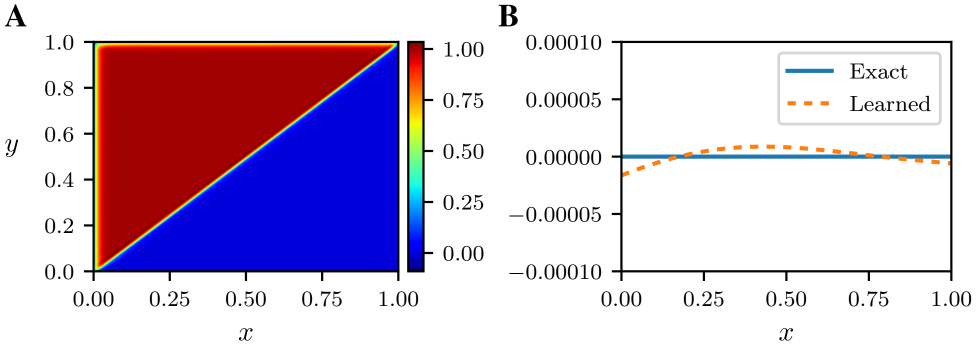

Figure S12: Boundary layer. Learned Green's function (A) and homogeneous solution (B) to a differential equation with a boundary layer around $x = 0$.

6 Differential operators in two dimensions

We demonstrate the ability of our method to learn Green's functions of two-dimensional operators by repeating the numerical experiment of [19], which consists of learning the Green's function of the Poisson operator on the unit disk $\Omega = D(0, 1)$, with homogeneous Dirichlet boundary conditions:

$$\mathcal{L}u = \nabla^2 u, \qquad u_{|\partial D(0,1)} = 0.$$

This experiment is a good benchmark for PDE learning techniques as the analytical expression of the Green's function in Cartesian coordinates can be expressed as [20]:

$$G_{\text{exact}}(x, y, \tilde{x}, \tilde{y}) = \frac{1}{4\pi} \ln\left(\frac{(x - \tilde{x})^2 + (y - \tilde{y})^2}{(x\tilde{y} - \tilde{x}y)^2 + (x\tilde{x} + y\tilde{y} - 1)^2}\right),$$

where $(x, y), (\tilde{x}, \tilde{y}) \in D(0, 1)$.

The training dataset for this numerical example is created as follows. First, we generate $N = 100$ random forcing terms using the command `randnfundisk` of the Chebfun software [4, 21, 22] with a frequency parameter of $\lambda = 0.2$, and then solve the Poisson equation, with corresponding right-hand sides, using a spectral method. Then, the forcing terms and system responses (*i.e.* solutions) are sampled at the $N_u = N_f = 673$ nodes of a disk mesh, generated using the Gmsh software [23]. The spatial discretization of the mesh is chosen to approximatively match the discretization ($N_u = N_f = 625$) of the Stokes example in the main text. Moreover, the mesh structure ensures that the repartition of the sample points is approximately uniform in the disk (Fig. S13C) and that the boundary is accurately captured.

The Green's function and homogeneous rational NNs have four hidden layers and width of 50 neurons, with 4 and 2 input nodes, respectively, as the Green's function is defined on $\Omega \times \Omega$. The two-dimensional integrals of the loss function (3) are discretized using uniform quadrature weights: $w_i = \pi/N_f$ for $1 \leq i \leq N_f$. In Fig. S13D to G, we visualize four, two-dimensional, slices of the learned Green's function together with two slices of the exact Green's function in panels A and B. Because of the symmetry in the Green's function, due to the self-adjointness of $\mathcal{L}$ and the boundary constraints, the exact Green's function satisfies $G(x, y, 0, 0) = G(0, 0, x, y)$ for $(x, y) \in D(0, 1)$. Therefore, we compare Fig. S13A to Fig. S13D, E, and similarly for Fig. S13B and Fig. S13F, G. We observe that the Green's function is accurately learned by the rational NN, which preserves low approximation errors near the singularity at $(x, y) = (\tilde{x}, \tilde{y})$, contrary to the Neural operator technique [19]. The visual artifacts present in Fig. S13E to G are likely due to the low spatial discretization of the training data.

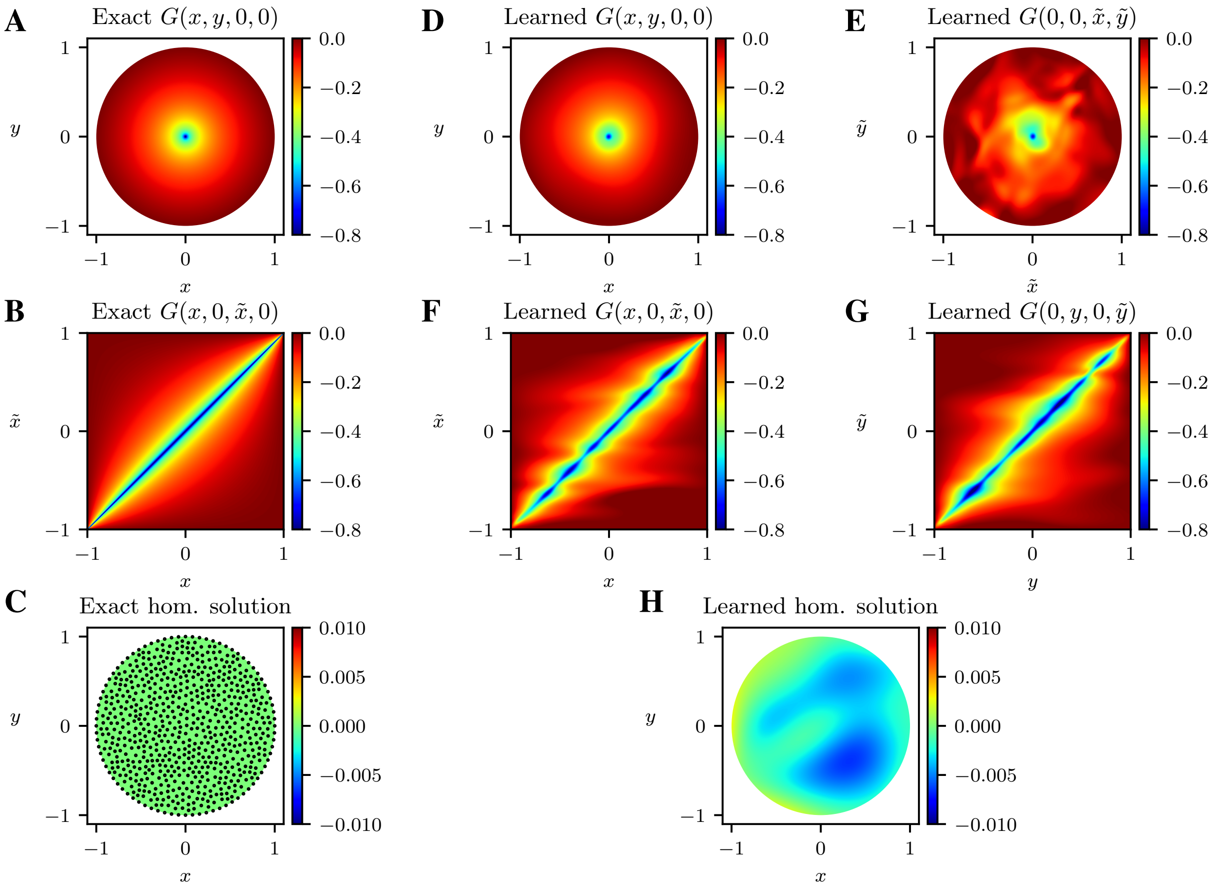

Figure S13: Poisson equation on the disk. Exact (A and B) and learned (D to F) Green's function of the Poisson operator on the unit disk, evaluated at two-dimensional slices. The colorbar is scaled to remove the singularity of the Green's function at $(x, y) = (\tilde{x}, \tilde{y})$. (C) Exact homogeneous solution with sample points for the training functions and (H) homogeneous solution learned by the rational NN.

7 System of differential equations

The method for discovering Green's functions of scalar differential operators extends naturally to systems of differential equations. Let $f = \begin{bmatrix} f^1 & \cdots & f^{n_f} \end{bmatrix}^\top : \Omega \to \mathbb{R}^{n_f}$ be a vector of n_f forcing terms and $u = \begin{bmatrix} u^1 & \cdots & u^{n_u} \end{bmatrix}^\top : \Omega \to \mathbb{R}^{n_u}$ be a vector of n_u system responses such that

$$\mathcal{L}\begin{bmatrix} u^1 \\ \vdots \\ u^{n_u} \end{bmatrix} = \begin{bmatrix} f^1 \\ \vdots \\ f^{n_f} \end{bmatrix}, \qquad D\left(\begin{bmatrix} u^1 \\ \vdots \\ u^{n_u} \end{bmatrix}, \Omega\right) = \begin{bmatrix} g^1 \\ \vdots \\ g^{n_u} \end{bmatrix}. \tag{11}$$

The solution to Eq. (11) with $f = 0$ is called the homogeneous solution and denoted by $u_{\text{hom}} = \begin{bmatrix} u^1_{\text{hom}} & \cdots & u^{n_u}_{\text{hom}} \end{bmatrix}^\top$. Similarly to the scalar case, we can express the relation between the system's response and the forcing term using Green's functions and an integral formulation as

$$u^i(x) = \sum_{j=1}^{n_f} \int_\Omega G_{i,j}(x,y) f^j(y)\,\mathrm{d}y + u^i_{\text{hom}}(x), \qquad x \in \Omega, \tag{12}$$

for $1 \leq i \leq n_u$. Here, $G_{i,j} : \Omega \times \Omega \to \mathbb{R} \cup \{\pm\infty\}$ is a component of the *Green's matrix* for $1 \leq i \leq n_u$ and $1 \leq j \leq n_f$, which consists of a $n_u \times n_f$ matrix of Green's functions:

$$G(x,y) = \begin{bmatrix} G_{1,1}(x,y) & \cdots & G_{1,n_f}(x,y) \\ \vdots & \ddots & \vdots \\ G_{n_u,1}(x,y) & \cdots & G_{n_u,n_f}(x,y) \end{bmatrix}, \qquad x, y \in \Omega.$$

Following Eq. (12), we remark that the differential equations decouple, and therefore we can learn each row of the Green's function matrix independently. That is, for each row $1 \leq i \leq n_u$, we train n_f NNs to approximate the components $G_{i,1}, \ldots, G_{i,n_f}$, and one NN to approximate the ith component of the homogeneous solution, u^i_{hom}.

As an example, we consider the following system of ordinary differential equations (ODEs) on $\Omega = [-1, 1]$:

$$\frac{d^2u}{dx^2} - v = f^1, \tag{13a}$$

$$\frac{-d^2v}{dx^2} + xu = f^2, \tag{13b}$$

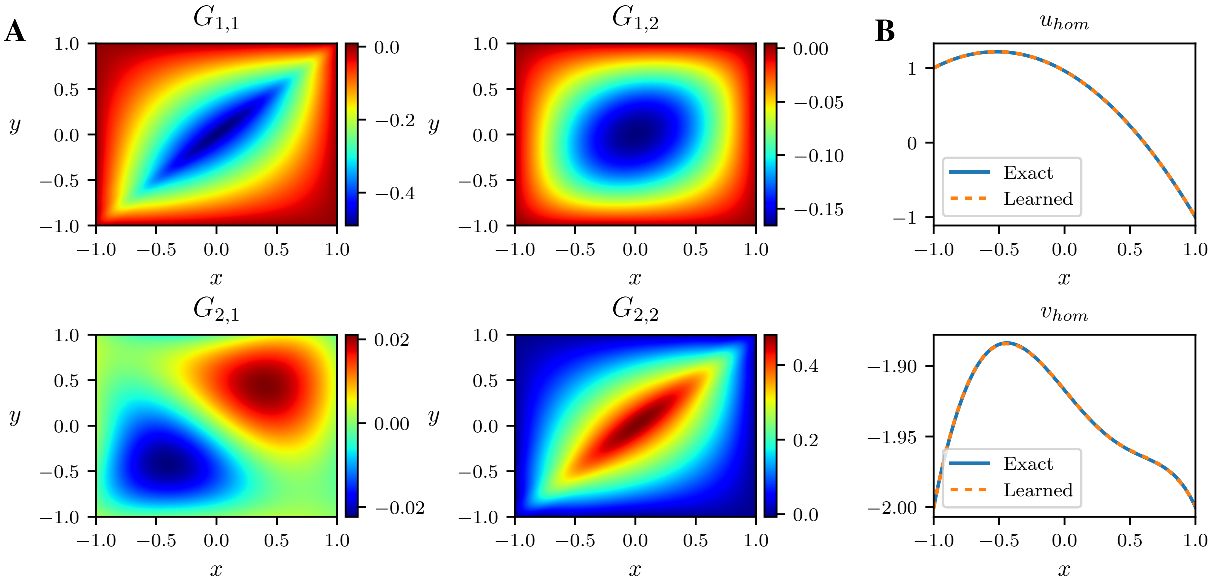

Figure S14: Green's matrix of system of ODEs. (A) Matrix of Green's function learned from the system of ordinary differential equations (13). (B) Homogeneous solutions associated with the system of ODEs.

with boundary conditions: $u(-1) = 1$, $u(1) = -1$, $v(-1) = v(1) = -2$. In Fig. S14, we display the different components of the Green's matrix and the exact solution (computed by a spectral method), along with the learned homogeneous solutions. We find that the Green's function matrix provides insight on the coupling between the two system variables, u and v, as shown by the diagonal components $G_{1,2}$ and $G_{2,1}$ of the Green's matrix in Fig. S14A. Similarly, the components $G_{1,1}$ and $G_{2,2}$ are characteristic of diffusion operators, which appear in Eq. (13). In this case, the Green's matrix can be understood as a 2×2 block inverse [24] of the linear operator, $\mathcal{L}$.

8 Analysis of main text examples

This section describes the implementation of the main text examples illustrated in Figs. 3 and 4.

8.1 Viscous shock

We first consider the following second-order differential operator [25] on $\Omega = [-1, 1]$:

$$\mathcal{L}u = 10^{-3}\frac{d^2u}{dx^2} + 2x\frac{du}{dx}, \qquad u(-1) = -1,\, u(1) = 1.$$

The robustness of our DL method with missing data in the vicinity of a shock front is analyzed in the panels D to F of Fig. 3, by applying the experiment of Section 4.5. We then remove the measurement points in the interval $[-0.2, 0.2]$ and train the Green's function and homogeneous NNs on this new dataset. The trained NNs are sampled on the whole domain to observe the generalization ability of our DL method.

8.2 Advection-diffusion on the right of the domain

Fig. 3G to I illustrates the learned Green's function and homogeneous solution to the differential operator:

$$\mathcal{L}u = 0.1\frac{d^2u}{dx^2} + \mathbb{I}_{(x\geq 0)}\frac{du}{dx}, \qquad u(-1) = 2,\, u(1) = -1,$$

on $\Omega = [-1, 1]$. Here, $\mathbb{I}_{(x\geq 0)}$ denotes the characteristic function on $x \geq 0$. The resulting equation is diffusive on the left half of the domain, while the advection is turned on for $x \geq 0$. We recognize the Green's function of the Laplacian operator, which is responsible for the diffusion behavior, within the visualization of the learned Green's function NN in Fig. 3G, restricted to the domain $[-1, 0] \times [-1, 0]$. Similarly, the upper right domain of the Green's function is characteristic of an advection-dominated behavior (Fig. S12A).

8.3 Linearized models of nonlinear operators

We emphasize that our DL method can be used to linearize and extract Green's functions from nonlinear boundary value problems of the form

$$\mathcal{L}u + \epsilon\mathcal{N}(u) = f, \qquad \mathcal{D}(u, \Omega) = g,$$

where $\mathcal{L}$ denotes a linear operator, $\mathcal{N}$ is a nonlinear operator, and $\epsilon < 1$ is a small parameter controlling the nonlinearity. We demonstrate this ability on the three nonlinear boundary value problems, dominated by the linearity, used in [26].

Fig. 4A of the main text illustrates the learned Green's function of a cubic Helmholtz system on $\Omega = [0, 2\pi]$ with homogeneous Dirichlet boundary conditions:

$$\frac{d^2u}{dx^2} + \alpha u + \epsilon u^3 = f(x),$$

where $\alpha = -1$ and $\epsilon = 0.4$. Next, in Fig. 4B, we consider a nonlinear Sturm–Liouville operator of the form:

$$[-p(x)u']' + q(x)(u + \epsilon u^3) = f(x), \qquad u(0) = u(2\pi) = 0,$$

with $p(x) = 0.4\sin(x) - 3$, $q(x) = 0.6\sin(x) - 2$, and $\epsilon = 0.4$. The notation u' denotes the derivative with respect to x, du/dx. Finally, the example represented in Fig. 4C is the learned Green's function of a nonlinear biharmonic operator:

$$[-p(x)u'']'' + q(u + \epsilon u^3) = f(x), \qquad u(0) = u(2\pi) = 0,$$

where $p = -4$, $q = 2$, and $\epsilon = 0.4$.

8.4 Lid-driven cavity problem

We consider a classical benchmark in fluid dynamics consisting of a two-dimensional lid-driven cavity problem [27]. We aim to discover the matrix of Green's functions of the Stokes flow [28], which is modelled by the following system of equations on the domain $\Omega = [0, 1]^2$,

$$\mu\nabla^2\mathbf{u} - \nabla p = \mathbf{f},$$

$$\nabla \cdot \mathbf{u} = 0.$$

Here, $\mathbf{u} = (u_x, u_y)$ is the fluid velocity, p is the pressure, $\mathbf{f} = (f_x, f_y)$ is an applied body force (*i.e.* a forcing term), and $\mu = 1/100$ is the dynamic viscosity. The fluid velocity satisfies

no-slip boundary conditions on the walls, except on the top wall where $\mathbf{u} = (1, 0)$. We first generate one hundred forcing terms, $\mathbf{f}$, with two smooth random components, in the Chebfun software [4, 21] using the `randnfun2` command with wavelength parameter $\lambda = 0.1$. The Stokes equations are then discretized with Taylor–Hood finite elements [29, 30] for the velocity and pressure on a mesh with 96×96 square cells and subsequently solved using the Firedrake finite element library [31]. We illustrate in Fig. S15 an example of applied body force and velocity solution obtained by solving the system of PDEs. We then create the training dataset for the NNs by sampling the applied body forces and corresponding velocity solutions, $\mathbf{u}$, on a regular 25×25 grid. The four Green's functions and two homogeneous NNs have the same architecture as the one described in the *Methods*, except that they have respectively four and two input nodes (instead of two and one) due to the current spatial dimension.

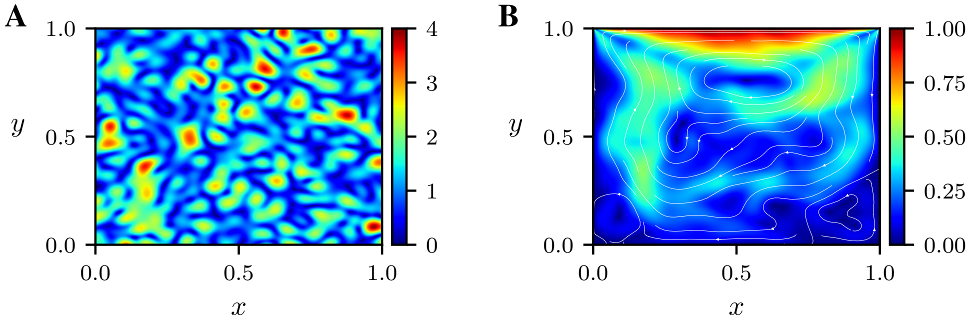

Figure S15: Training functions for Stokes flow. (A) Magnitude of a random applied body force used as a forcing term in the Stokes equations. (B) Velocity magnitude and streamlines of the system's response.

The four components of the Green's matrix for the Stokes flow are evaluated on the two-dimensional slice $(x, y, 0.5, 0.5)$, for $x, y \in [0, 1]$, and displayed in Fig. 4D of the main text. This figure allows us to visualize the system's response to a point force, $\mathbf{f} = (f_x, f_y)$, located at

$(0.5, 0.5)$, with the system's response being denoted as $\mathbf{u} = (u_x, u_y)$, where

$$u_x(x, y) = G_{1,1}(x, y, 0.5, 0.5)f_x + G_{1,2}(x, y, 0.5, 0.5)f_y,$$

$$u_y(x, y) = G_{2,1}(x, y, 0.5, 0.5)f_x + G_{2,2}(x, y, 0.5, 0.5)f_y,$$

for $x, y \in [0, 1]$. The visualization of the $G_{2,2}$ component in Fig. 4D, corresponding to the system's response to a unitary vertical point force $\mathbf{f} = (0, 1)$ is reminiscent of Fig. 1 of [32].

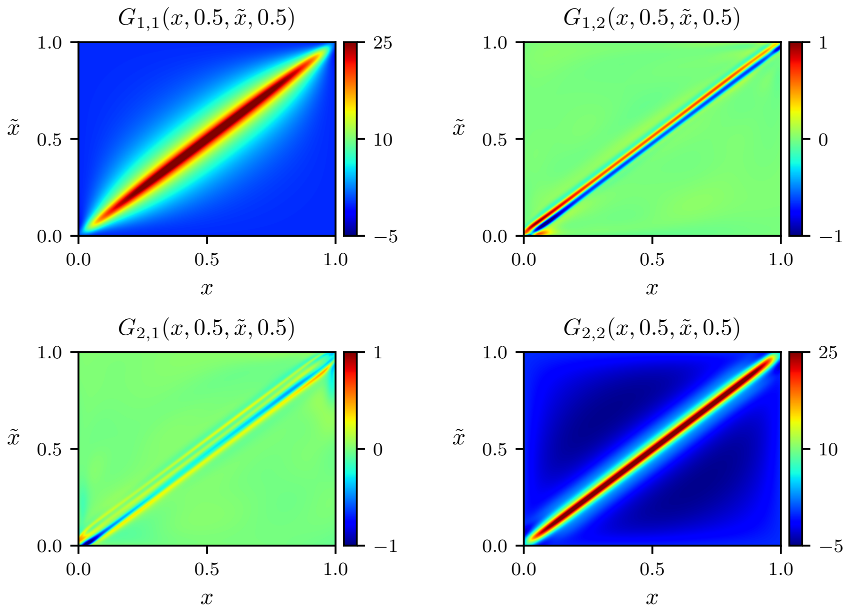

Figure S16: 2nd Green's matrix slice of Stokes flow. The four components of the Green's matrix learned by a rational neural network evaluated at the two-dimensional slice $(x, 0.5, \tilde{x}, 0.5)$.

Finally, we evaluate the components of the Green's matrix at three other two-dimensional slices: $(x, 0.5, \tilde{x}, 0.5)$, $(0.5, y, 0.5, \tilde{y})$, $(0.5, 0.5, \tilde{x}, \tilde{y})$ and display them respectively in Figs. S16 to S18. These figures illustrate the different symmetries of the Green's matrix, which are captured by the rational NNs. As an example, we see in Figs. S16 and S17 that $G_{1,1}(x, 0.5, \tilde{x}, 0.5) = G_{2,2}(0.5, x, 0.5, \tilde{x})$ and $G_{2,2}(x, 0.5, \tilde{x}, 0.5) = G_{1,1}(0.5, x, 0.5, \tilde{x})$, for $x, \tilde{x} \in [0, 1]$. Similarly, we find in Fig. S18 that $G_{1,1}(0.5, 0.5, \tilde{x}, \tilde{y}) = G_{1,1}(0.5, 0.5, \tilde{y}, \tilde{x})$ and $G_{1,2}(0.5, 0.5, \tilde{x}, \tilde{y}) =$

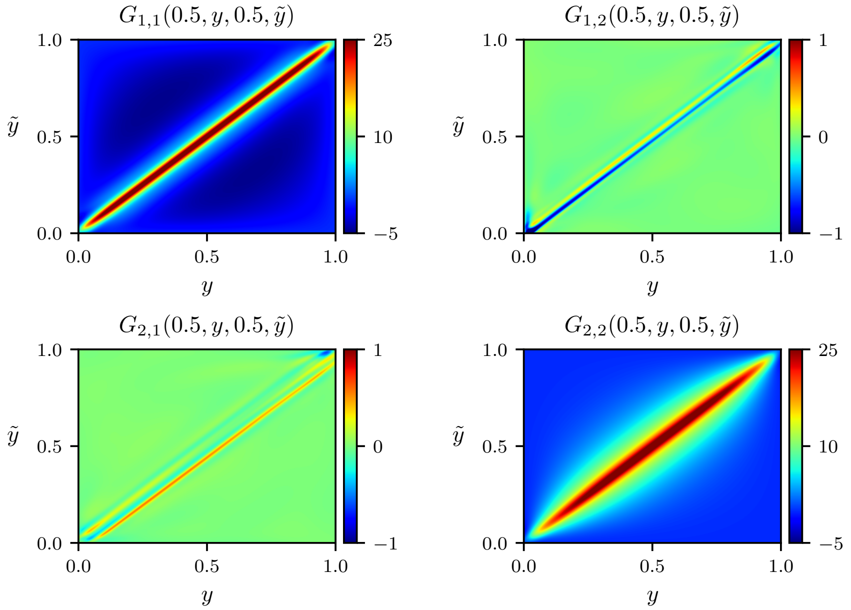

Figure S17: 3rd Green's matrix slice of Stokes flow. The four components of the Green's matrix learned by a rational neural network evaluated at the two-dimensional slice $(0.5, y, 0.5, \tilde{y})$.

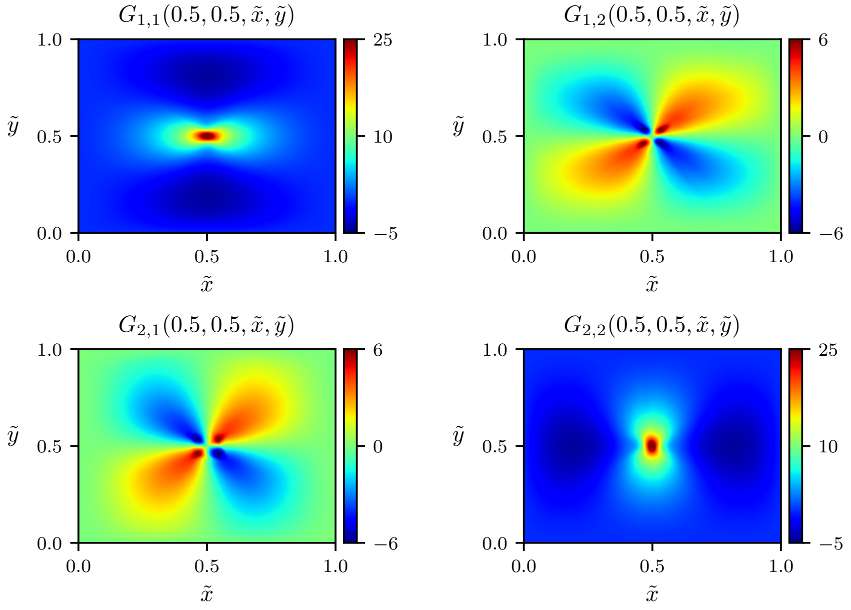

Figure S18: 4th Green's matrix slice of Stokes flow. The four components of the Green's matrix learned by a rational neural network evaluated at the two-dimensional slice $(0.5, 0.5, \tilde{x}, \tilde{y})$.

$G_{2,1}(0.5, 0.5, \tilde{x}, \tilde{y})$, for $\tilde{x}, \tilde{y} \in [0, 1]$. The $G_{1,2}$ and $G_{2,1}$ components of the Green's matrix in Fig. S16 highlight a singularity along the diagonal $(x, 0.5, x, 0.5)$ for $x \in [0, 1]$. However, this singularity does not prevent the rational NNs from accurately learning the different components of the Green's matrix displayed in Fig. 4D and Figs. S16 to S18.

9 Time-dependent equations

In this section, we show that one can use a time-stepping scheme to discretize a time-dependent PDE and learn the Green's function associated with the time-propagator operator $\tau : u_n \to u_{n+1}$, where u_n is the solution of the PDE at time $t = n\Delta t$ for a fixed time step Δt. As an example, we consider the time-dependent Schrödinger equation with a harmonic trap potential $V(x) = x^2$ given by

$$i\frac{\partial \psi(x,t)}{\partial t} = -\frac{1}{2}\frac{\partial^2 \psi(x,t)}{\partial x^2} + x^2\psi(x,t), \qquad x \in [-3, 3], \tag{14}$$

with homogeneous Dirichlet boundary conditions. We use a Crank–Nicolson time-stepping scheme with time step $\Delta t = 2 \times 10^{-2}$ to discretize Eq. (14) in time and obtain

$$i\frac{\psi_{n+1} - \psi_n}{\Delta t} = \frac{1}{2}\left[-\frac{1}{2}\frac{d^2\psi_{n+1}}{dx^2} + x^2\psi_{n+1} - \frac{1}{2}\frac{d^2\psi_n}{dx^2} + x^2\psi_n\right].$$

Our training dataset consists of one hundred random initial forcing functions ψ_n at time t and associated response ψ_{n+1} at time $t + \Delta t$. The functions ψ_n have real and imaginary parts sampled from a Gaussian process with periodic kernel and length-scale parameter $\lambda = 0.5$ (see Section 1), and multiplied by the Gaussian damping function $g(x) = e^{-x^6/20}$ to ensure that the functions decay to zero before reaching the domain boundaries. We then train a rational neural network to learn the Green's function G associated with the time-propagator operator such that

$$\tau(\psi_n)(x) = \int_{-3}^{3} G(x,y)\psi_n(y)\,\mathrm{d}y = \psi_{n+1}(x), \qquad x \in [-3, 3].$$

Note that since ψ takes complex values, we in fact split Eq. (14) into a system of equations for the real and imaginary parts of ψ, and learn the Green's matrix associated with the system (see Section 7).

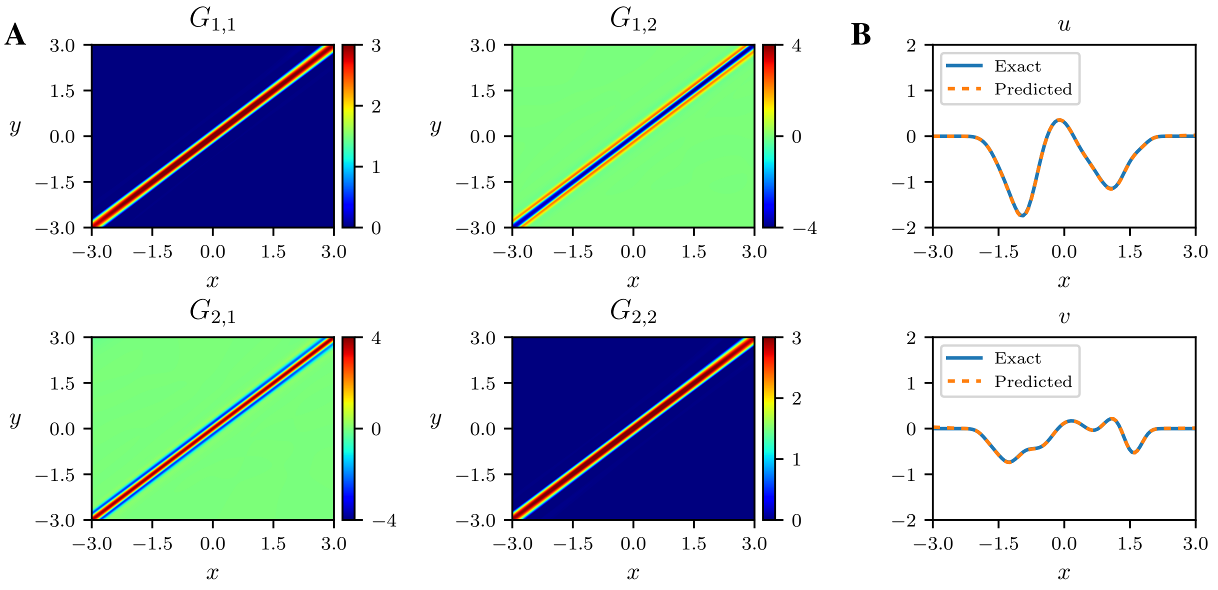

Figure S19: Green's matrix of the time-dependent Schrödinger equation. (A) The four components of the Green's matrix for the time propagator operator of the time-dependent Schrödinger equation discretized using a time-stepping scheme. (B) Real and imaginary components of the worst case prediction of the solution at the next time step.

We report the Green's matrix of the time-propagator operator for the Schrödinger equation in Fig. S19A and observe that the four components are dominated by the diagonal, which is expected for a small time-step. Additionally, we evaluate the accuracy of the learned Green's functions by generating a testing dataset with one hundred initial functions ψ_n, sampled from the same distribution, and associated solution ψ_{n+1} at time $t+\Delta t$. We then compute the average (over the one hundred test cases) relative error in the L^2 norm between the exact solution ψ_{n+1} and the one predicted using the learned Green's functions, ψ_{n+1}^{pred}, as

$$\text{relative error} = \|\psi_{n+1} - \psi_{n+1}^{\text{pred}}\|_{L^2([-3,3])} / \|\psi_{n+1}\|_{L^2([-3,3])},$$

where ψ_{n+1}^{pred} is defined as

$$\psi_{n+1}^{\text{pred}}(x) = \int_{-3}^{3} G(x,y)\psi_n(y)\,\mathrm{d}y, \qquad x \in [-3,3].$$

Finally, we obtain an average relative error of 1.3% with standard deviation 0.2% across the 100 test cases, confirming the good accuracy of our method. We display the worst-case prediction of the solution ψ_{n+1} in Fig. S19B.

References

[1] Rasmussen, C. E. & Williams, C. *Gaussian processes for machine learning* (MIT Press, 2006).

[2] Boullé, N. & Townsend, A. Learning elliptic partial differential equations with randomized linear algebra. *Found. Comput. Math.* (2022).

[3] Trefethen, L. N. *Spectral Methods in MATLAB* (SIAM, 2000).

[4] Driscoll, T. A., Hale, N. & Trefethen, L. N. *Chebfun Guide* (Pafnuty Publications, 2014). URL http://www.chebfun.org/docs/guide/.

[5] Süli, E. & Mayers, D. F. *An Introduction to Numerical Analysis* (Cambridge University Press, 2003).

[6] Binder, K. *et al. Monte Carlo Methods in Statistical Physics* (Springer Science & Business Media, 2012).

[7] Bengio, Y. Practical recommendations for gradient-based training of deep architectures. In *Neural networks: Tricks of the trade*, 437–478 (Springer, 2012).

[8] George, A. P. & Powell, W. B. Adaptive stepsizes for recursive estimation with applications in approximate dynamic programming. *Mach. Learn.* **65**, 167–198 (2006).

[9] Smith, L. N. Cyclical learning rates for training neural networks. In *IEEE Winter Conference on Applications of Computer Vision*, 464–472 (IEEE, 2017).

[10] Olver, P. J. *Applications of Lie groups to differential equations* (Springer-Verlag, 1993), 2 edn.

[11] Stakgold, I. & Holst, M. J. *Green's Functions and Boundary Value Problems* (John Wiley & Sons, 2011).

[12] Hsing, T. & Eubank, R. *Theoretical foundations of functional data analysis, with an introduction to linear operators* (John Wiley & Sons, 2015).

[13] Trefethen, L. N., Birkisson, A. & Driscoll, T. A. *Exploring ODEs* (SIAM, 2017).

[14] Trefethen, L. N., Nakatsukasa, Y. & Weideman, J. Exponential node clustering at singularities for rational approximation, quadrature, and PDEs. *Numer. Math.* **147**, 227–254 (2021).

[15] Stahl, H. Best uniform rational approximation of $|x|$ on $[-1, 1]$. *Mat. Sb.* **183**, 85–118 (1992).

[16] Stahl, H. Best uniform rational approximation of x^α on $[0, 1]$. *Bull. Am. Math. Soc.* **28**, 116–122 (1993).

[17] Beyene, W. T. Pole-clustering and rational-interpolation techniques for simplifying distributed systems. *IEEE T. Circuits-I* **46**, 1468–1472 (1999).

[18] Wegert, E. *Visual Complex Functions: An Introduction with Phase Portraits* (Springer Science & Business Media, 2012).

[19] Li, Z. *et al.* Neural operator: Graph kernel network for partial differential equations. *arXiv preprint arXiv:2003.03485* (2020).

[20] Myint-U, T. & Debnath, L. *Linear Partial Differential Equations for Scientists and Engineers* (Birkhäuser Basel, 2007).

[21] Filip, S., Javeed, A. & Trefethen, L. N. Smooth random functions, random ODEs, and Gaussian processes. *SIAM Rev.* **61**, 185–205 (2019).

[22] Wilber, H., Townsend, A. & Wright, G. B. Computing with functions in spherical and polar geometries II. The disk. *SIAM J. Sci. Comput.* **39**, C238–C262 (2017).

[23] Geuzaine, C. & Remacle, J.-F. Gmsh: A 3-D finite element mesh generator with built-in pre-and post-processing facilities. *Int. J. Numer. Methods Eng.* **79**, 1309–1331 (2009).

[24] Lu, T.-T. & Shiou, S.-H. Inverses of 2×2 block matrices. *Comput. Math. Appl.* **43**, 119–129 (2002).

[25] Lee, J.-Y. & Greengard, L. A fast adaptive numerical method for stiff two-point boundary value problems. *SIAM J. Sci. Comput.* **18**, 403–429 (1997).

[26] Gin, C. R., Shea, D. E., Brunton, S. L. & Kutz, J. N. DeepGreen: deep learning of Green's functions for nonlinear boundary value problems. *Sci. Rep.* **11**, 1–14 (2021).

[27] Elman, H. C., Silvester, D. J. & Wathen, A. J. *Finite Elements and Fast Iterative Solvers: With Applications in Incompressible Fluid Dynamics* (Oxford University Press, 2014), 2nd edn.

[28] Blake, J. R. A note on the image system for a Stokeslet in a no-slip boundary. *Math. Proc. Camb. Philos. Soc.* **70**, 303–310 (1971).

[29] Boffi, D., Brezzi, F. & Fortin, M. *Mixed Finite Element Methods and Applications* (Springer, 2013).

[30] Taylor, C. & Hood, P. A numerical solution of the Navier-Stokes equations using the finite element technique. *Comput. Fluids* **1**, 73–100 (1973).

[31] Rathgeber, F. *et al.* Firedrake: automating the finite element method by composing abstractions. *ACM Trans. Math. Softw.* **43**, 1–27 (2016).

[32] Ekiel-Jeżewska, M., Boniecki, R., Bukowicki, M. & Gruca, M. Stokes velocity generated by a point force in various geometries. *Eur. Phys. J. E* **41**, 1–7 (2018).
